# Safety and efficacy of Igk-TATk-CDKL5 gene therapy in mosaic CDKL5 deficiency

**DOI:** 10.1016/j.neurot.2025.e00727

**Published:** 2025-09-02

**Authors:** Giorgio Medici, Marianna Tassinari, Manuela Loi, Angelica Marina Bove, Beatrice Casadei Garofani, Greta Volpedo, Nicola Mottolese, Gabriele Matteoli, Viviana Lo Martire, Chiara Berteotti, Giulia Candini, Federica Trebbi, Antonella Riva, Pasquale Striano, Giovanna Zoccoli, Giulia Curia, Stefania Trazzi, Elisabetta Ciani

**Affiliations:** a Department of Biomedical and Neuromotor Sciences, University of Bologna, Italy; b Department of Biomedical, Metabolic and Neural Sciences, University of Modena and Reggio Emilia, Modena, Italy; c Department of Neurosciences, Rehabilitation, Ophthalmology, Genetics, Maternal and Child Health (DINOGMI), University of Genoa, Genoa, Italy; d IRCCS Istituto Giannina Gaslini, Genoa, Italy

**Keywords:** AAV gene therapy, Igk-TATk-CDKL5, Cross-correction, Heterozygous *Cdkl5* female mice, Brain disorder

## Abstract

CDKL5 Deficiency Disorder (CDD) is a severe neurodevelopmental disorder caused by mutations in the X-linked CDKL5 gene, resulting in early-onset seizures, developmental delays, and cognitive and sensorimotor impairments. While emerging therapies show promise, substantial challenges remain in developing a cure for CDD. In our prior work, we developed an innovative gene therapy strategy based on an Igk-TATk-CDKL5 fusion protein, which enhances brain distribution of the therapeutic protein, significantly improving treatment efficacy in a *Cdkl5* knockout male mouse model. However, CDKL5 dosage sensitivity may pose challenges in patients with mosaic loss of CDKL5 function, potentially limiting the treatment's effectiveness or even exacerbating clinical symptoms. In this study, we aimed to address this gap by evaluating the safety and efficacy of Igk-TATk-CDKL5 therapy in a heterozygous female mouse model (*Cdkl5* +/−), which better represents the majority of human CDD patients. We found that introducing Igk-TATk-CDKL5 significantly improved behavioral phenotypes and corrected brain structural defects, such as dendritic morphology and connectivity. Importantly, no adverse effects were observed in the brain or peripheral organs (e.g., the heart), indicating that CDKL5 overexpression in the heterozygous condition was well tolerated. These findings support the therapeutic potential of Igk-TATk-CDKL5 and suggest that a possible cross-correction mechanism may contribute to its efficacy, even in the context of mosaic CDKL5 deficiency. This approach may therefore offer promising therapeutic outcomes for patients with CDD.

## Graphical abstract

**Figure:**
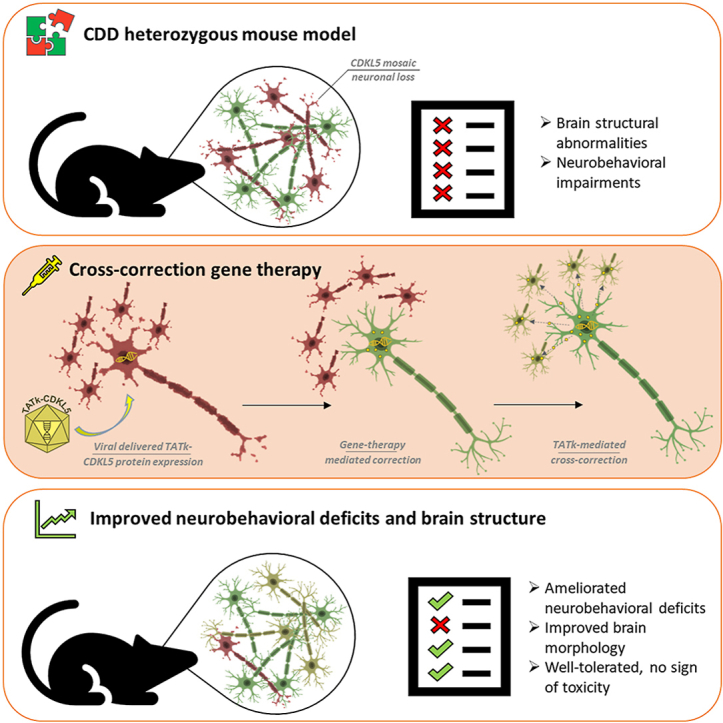

## Introduction

CDKL5 (cyclin-dependent kinase-like 5) Deficiency Disorder (CDD) is a severe X-linked neurodevelopmental disorder caused by CDKL5 kinase misfunction that is due to mutations in the *CDKL5* gene [[1], [2], [3]]. CDKL5 deficiency results in a severe, chronically debilitating disorder, characterized by early-onset epileptic seizures, intellectual disability, sensorimotor impairment, and visual deficits [[4], [5], [6]]. Although hemizygous males have also been reported, most CDKL5 patients are females who are heterozygous for CDKL5 deficiency with mosaic CDKL5 expression at the cellular level due to random X-chromosome inactivation [7,8]. There is currently no cure or effective treatment to ameliorate the cognitive and behavioral symptoms of CDD. Several mouse models of CDD, primarily *Cdkl5* knockout (KO) mice, have been generated to study the disease [[9], [10], [11], [12], [13]]. These models mimic some clinical CDD manifestations [[9], [10], [11],^14^,^15^], including severe learning and memory impairments, autistic-like behaviors, motor stereotypies, and visual impairment; however, spontaneous seizures were not reported in these independently generated constitutive *Cdkl5* KO mice [[9], [10], [11]]. Interestingly, evidence of spontaneous seizure activity has recently been described only in heterozygous *Cdkl5* +/− female mice [16,17], which, therefore, recapitulate features of epilepsy in CDD.

Treatment approaches for monogenic diseases have predominantly centered on complementing affected cells with a functional protein or gene copy. A preclinical study on gene replacement therapy in CDD was published in 2020 [18]. Authors showed a modest therapeutic effect of gene therapy with CDKL5 in the *Cdkl5* KO mouse [18]. This low efficacy was attributed to the necessity of a more robust brain transduction to ameliorate behavioral deficits in this mouse model [19]. Similarly, intracerebroventricular injection of very high viral loads (5 × 10^11^ vg/g brain) into neonatal mice resulted in only partial rescue of the behavioral deficits observed in *Cdkl5* KO models [20], suggesting that broad and efficient CDKL5 replacement is critical for therapeutic success. To overcome the limitations posed by the restricted number of transduced cells in the CNS and the safety concerns associated with the administration of high viral doses, we recently developed a virally delivered transgene encoding a modified CDKL5 protein engineered for secretion and cell penetration [21]. Specifically, we design a vector expressing an Igk-TATk-CDKL5 fusion protein which can be secreted via constitutive secretory pathways and internalized by neighboring cells due to the cell-penetration capability of the TATk peptide. Upon viral transduction, cells not only produce the therapeutic CDKL5 protein, but also release it into the extracellular space, enabling uptake by non-transduced cells through a cross-correction mechanism. Using this strategy, we achieved a broader biodistribution of CDKL5 across the mouse brain. Notably, gene therapy based on cross-correction proved to be significantly more effective in ameliorating brain defects in male *Cdkl5* KO mice compared to the conventional gene replacement approach relying solely on native CDKL5 [21].

While this gene therapy approach offers a promising strategy for the treatment of CDD, a critical gap remains in understanding its therapeutic potential under clinically relevant conditions. In particular, its efficacy and safety have not yet been evaluated in the context of mosaic loss of CDKL5, which represents the typical condition of most CDD patients who are heterozygous for mutations in the X-linked CDKL5 gene. The use of such treatments may be limited by findings reporting neurobehavioral alterations in patients with duplications of genomic regions containing the CDKL5 gene, suggesting that an increase in CDKL5 expression level might be pathogenic [[22], [23], [24]]. Although the clinical data are difficult to interpret, the presence of a dosage-sensitivity for CDD is plausible and must be taken into consideration. Thus, it remains to be elucidated whether the additional expression of an exogenous CDKL5 protein in wild-type cells would lead to an abnormal or pathogenic phenotype due to CDKL5 overexpression toxicity.

Here, by exploiting the heterozygous *Cdkl5* KO female mouse, we assessed the efficacy of a cross-correction-based gene therapy strategy in restoring mouse brain structural abnormalities and, consequently, behavioral outcomes. Furthermore, we evaluated the safety profile of this approach by investigating the effect of CDKL5 overexpression in brain cells expressing the endogenous native form of CDKL5 and in peripheral tissues with physiologically low CDKL5 expression levels, as well as in terms of preservation of normal wake-sleep behavior, which represents an index of health status for the animals.

## Material and Methods

### Production of AAVPHP.B_Igk-TATk-CDKL5 vector

To express the Igk-TATk-CDKL5 protein via AAV-mediated gene delivery, we used the pAAV_CBh-Igk-TATk-CDKL5-HA plasmid previously described [21]. Briefly, the Igk-TATk-hCDKL5 expression cassette was subcloned into the backbone of pAAV-CBh-DIO-EGFP (plasmid #87 168, Addgene), replacing the EGFP cassette. The vector genome, flanked by AAV2 inverted terminal repeats (ITRs), comprises: 1 - CBh promoter (0.8 kbp) for robust, ubiquitous expression; 2 - Igk secretion signal fused to the TATk cell-penetrating peptide (sequence: MetETDTLLLWVLLLWVPGSTGDAAQP ARRARRTKLAAYARKAARQARA); 3 - hCDKL5 open reading frame (total length 3.1 kbp, including leader); 4 - WPRE element for enhanced transcript stability; 5 - SV40 polyadenylation signal (0.4 kbp). A C-terminal haemagglutinin (HA) epitope tag was appended for immunodetection. AAVPHP.B expressing the Igk-TATk-CDKL5 protein was produced by Innovavector srl (Pozzuoli (NA), Italy).

### Animal husbandry

The mice used in this work derive from the *Cdkl5* null strain in the C57BL/6 N background developed in Ref. [10] and backcrossed in C57BL/6J for three generations. Animals were genotyped as previously described [10]. *Cdkl5* +/− heterozygous female mice were produced by crossing *Cdkl5* +/− females with *Cdkl5* +/Y males, and littermate controls were used for all experiments. P0, postnatal day zero, was designated as the day of birth, and 24 h later mice were considered as 1-day-old animals (P1). After weaning (P21–23), mice were housed three to five per cage on a 12-h light/dark cycle in a temperature- and humidity-controlled environment, with food and water provided ad libitum. The animals’ health and comfort were controlled by the veterinary service. The study protocols complied with EU Directive 2010/63/EU and with Italian law (DL 26, March 4, 2014) and were approved by the Italian Ministry of Health (protocol n° 535/2022-PR). In this study, all efforts were made to minimize animal suffering and to keep the number of animals used to a minimum. Experiments were carried out on a total of 52 *Cdkl5* KO mice (*Cdkl5* +/+, n = 22; *Cdkl5* +/−, n = 31).

### In vivo AAV delivery

Ten-month-old mice were administered with AAVPHP.B_Igk-TATk-CDKL5 vector at a dose of 10^12^ viral genome (vg)/mouse via intracarotid injection following the surgical procedure previously described in Ref. [21]. Untreated age-matched *Cdkl5* +/− and wild-type (+/+) mice were used as controls to assess the effects of gene therapy.

### Behavioral assays

Behavioral tests were performed 2 months after the intracarotid infusion. All animal behavioral studies and analyses were performed blinded to genotype and treatment. The sequence of the tests was arranged to minimize the possibility of one test influencing the subsequent evaluation of the next test, and mice were allowed to recover for 2–3 days between different tests. Mice were allowed to habituate to the testing room for at least 1 hour before the test, and testing was performed at the same time of day.

#### Nesting

Nest building ability was evaluated as proposed by Ref. [25]. Animals were placed in individual cages with standard bedding, and a standard piece of paper towel (23 × 23 cm) was provided. The nests were independently assessed at 24 hours by two trained operators using the following scoring system: 0 - no nest, 1 - primitive flat nest (pad shaped, consisting of a flat paper tissue which slightly elevates a mouse above the bedding), 2 - more complex nest (including warping and biting the paper towel), 3 - complex accurate cup-shaped nests (with shredded paper interwoven to form the walls of the cup), and 4 - complex hooded nest, with walls forming a ceiling so the nest becomes a hollow sphere with one opening.

#### Marble burying

The marble burying test was performed by placing animals individually in a home-cage-like environment with 5 cm of unscented standard bedding material and 20 marbles (14.3 mm in diameter) arranged in a 4 × 5 matrix; the animals were left undisturbed for 30 minutes. The number of marbles that were at least two-thirds buried at the end of the trial was counted.

#### Hind-limb clasping

Animals were suspended by their tail for 2 minutes and hind-limb clasping time was assessed from video recordings. A clasping event was defined by the retraction of limbs toward the body and midline.

#### Open field test

To assess locomotion, the animals were placed in the center of a square arena (50 × 50 cm) and their behavior was monitored for 15 minutes using a video camera placed above the center of the arena. Distinct features of locomotor activity, including total distance traveled, and average locomotion velocity were scored using EthoVision15XT software (Noldus, Wageningen, The Netherlands). The average locomotion velocity was calculated as the ratio between distance traveled and time. Animals exhibiting extreme stereotypical behavior (i.e., more than 100 repetitive jumps) were excluded from the locomotion analysis. The number of stereotypical jumps—defined as repetitive beam breaks occurring in less than 1 second—was manually counted by a trained observer. Test chambers were cleaned with 70 % ethanol between test subjects.

#### Accelerating rotarod assay

Before the first test session, animals were briefly trained at a constant speed of 5 rpm on the rotarod apparatus (Ugo Basile, Gemonio, Italy) for 30 seconds. Thirty minutes later, testing was performed at an accelerating linear speed (from 5 to 35 rpm over 270 seconds, followed by 30 seconds at maximum speed). Four testing trials with an intertrial interval of 1 hour were performed. The latency to fall and the number of passive rotations (i.e., rotations during which the mouse does not perform any coordinated movement but is passively transported by the rotating apparatus) were recorded for each trial.

#### Barnes maze

The test was used to measure spatial memory acquisition and retention. An escape box was placed under one of the holes on a circular platform (1 m in diameter) with 20 holes (each 5 cm in diameter) along the perimeter of the platform (Ugo Basile, Gemonio, Italy) that was elevated 60 cm above the floor. Mice were placed in a cylindrical chamber at the center of the maze and allowed to explore to locate the escape box. Mice were trained to find the escape box for 3 days (3 trials per day, max. 3 minutes per trial) with a 30 minute intertrial interval. To assess spatial memory retention, a 90 second probe trial with the escape box removed was performed 24 hours after the last trial. Latency to locate the escape box was recorded using a video tracking system (Ethovision 15XT, Noldus Information Technology, The Netherlands). Mice showing freezing behavior for more than two-thirds of the test duration were excluded from the analysis.

#### Mechanical allodynia test

Mechanical allodynia was quantified by measuring the hind paw withdrawal response to von Frey filament stimulation. After a 15-minute acclimation period in acrylic cages (12 × 20 × 17 cm) with a wire-grid floor (5 mm^2^), a single, un-bending filament was applied perpendicularly to the hind paw and the stimulus of increasing force was applied to the hind paw and ceased when the animal showed paw withdrawal. Clear paw withdrawal, shaking, or licking of the paw was considered as a nociceptive-like response. The mechanical threshold, defined as the force (g) responsible for paw withdrawal, was calculated using the average of three measurements taken from each paw [26].

### EEG/EMG implantation surgery and recordings

Female mice (wild-type +/+, n = 14; *Cdkl5* +/−, n = 13; *Cdkl5* +/− GT, n = 11) underwent surgical implantation of electrodes for differential frontal-parietal electroencephalographic (EEG) and electromyographic (EMG, neck muscles) recordings under general anesthesia (isoflurane 1.8–2.4 % in O_2_) and intraoperative analgesia (Carprofen, 0.1 mg s.c., Pfizer Italy, Latina, Italy), as previously described [27]. All electrodes were connected to a miniature, custom-built socket that was affixed to the skull using stainless-steel anchor screws (2.4 mm length, P1 Technologies, Roanoke, VA, USA), dental cement (RelyX Unicem, 3 M ESPE, Segrate, Milano, Italy), and dental acrylic (Respal NF, SPD, Mulazzano, Italy). Following surgery, mice were allowed to recover for 13–15 days.

After 4–6 days of habituation to the recording setup, baseline EEG/EMG recordings were carried out for 48 hours, starting at Zeitgeber Time 0 (ZT0), to assess wake-sleep behavior. In parallel, these biosignals were used to evaluate the epileptic phenotype in the same experimental animals. Recordings were performed in freely moving mice individually housed in their home cages [27], under a 12:12 h light-dark cycle, with ambient temperature maintained at 25 °C and free access to food and water. EEG and EMG signals were transmitted via cable; a rotating swivel (SL2+2C/SB, P1 Technologies, Roanoke, VA, USA) mounted on a balanced suspensor arm prevented cable twisting and compensated for its weight, allowing the mice unrestricted movement.

After baseline recordings, all mice underwent 6 hours of sleep deprivation beginning at ZT0, followed by 18 hours of sleep recovery. Sleep deprivation was used as a trigger for the induction of epileptic seizures and interictal activity [28], and it was conducted using gentle handling while continuously monitoring EEG/EMG signals in real-time. Specifically, whenever EMG tone decreased and was accompanied by slow delta EEG waves (0.5–4 Hz), the investigator gently tapped the cage or touched the mouse with a cotton swab to prevent sleep onset.

EEG and EMG signals were amplified and filtered (EEG: 0.3–100 Hz with a 50 Hz notch filter; EMG: 100–1000 Hz with a 50 Hz notch filter; 7P511J amplifiers, Grass, West Warwick, RI, USA), digitized at 16-bit resolution and 1024 Hz, and subsequently downsampled to 128 Hz for storage. Data acquisition was performed using custom software developed in LabVIEW (National Instruments, Austin, TX, USA). Video recordings were also captured during the baseline period using an infrared camera (Basler CMOS E2V Global Shutter B&W 1280x1024, 60 fps, PoE, C-Mount; Basler, Milano, Italy) and acquisition software (Pylon Viewer, Basler, Milano, Italy).

### Sleep analysis

Data were analyzed using MATLAB R2020a (MathWorks, Natick, MA, USA; www.mathworks.com). Wakefulness, non-rapid-eye-movement sleep (NREMS), and rapid-eye-movement sleep (REMS) were scored either automatically based on raw EEG and EMG signals (4-second epochs) using SCOPRISM, a validated algorithm [29], or manually through visual inspection of EEG and EMG recordings with 4-second resolution, as previously described in detail [30].

Sleep architecture was assessed using a threshold of 12 seconds (i.e., three consecutive 4-second epochs) to define the duration of wake-sleep episodes [30]. The sleep fragmentation index was calculated as the number of awakenings divided by total sleep time, in accordance with previous methods [30].

Spectral analysis of the EEG signal was performed on artifact-free 4-second epochs using a discrete Fourier transform. Slow-wave activity (SWA) during NREMS (δ frequency band, 1–4 Hz) and the EEG power spectrum during both NREMS and REMS were analyzed by identifying the peak frequency within each state [27]. Baseline SWA values were calculated in 2-hour temporal bins and expressed as a percentage of the mean SWA during NREMS between ZT0 and ZT4. EEG power spectra during NREMS and REMS were expressed as a percentage of the total EEG power in each respective state.

### Seizure analysis

Video-EEG analysis was performed offline by an experimenter blinded to genotype using LabChart (ADInstruments NZ Limited, Dunedin, NL). A 50 Hz Notch filter was applied offline. Ictal events were defined as a sudden change in amplitude greater than 2 times the standard deviation from the baseline mean and were detected based on high-frequency rhythmic activity (>2.5 Hz) lasting more than 10 seconds [31]. After the analysis of the EEG tracings, video recordings were analyzed starting from the moment in which the electrographic seizure appeared in EEG signals and a behavioral classification was performed according to a scale rearranged from Ref. [32] as follows: 0: no changes in behavior; 1: Facial or body automatisms, myoclonic jerks with sudden and repetitive movement of the head and neck with or without tail stiffening; 2: Atypical (unilateral or incomplete) clonic seizure; 3: Clonic seizure with forelimb clonus and rearing; 4: Tonic-clonic seizure with an initial wild run and subsequent loss of righting reflex; 5: Tonic-clonic seizure with full extension of fore and hind-limb. Seizure frequency was calculated by dividing the number of seizures by the total hours recorded for each phase (baseline and post-deprivation). Seizure frequency was calculated as the number of seizure-like events/h of recordings. Seizure duration was determined on EEG traces.

### Tissue dissection and collection

Animals were anesthetized with 2 % isoflurane (in pure oxygen) and sacrificed through cervical dislocation. Blood samples were collected for analysis of circulating cytokine levels. Hearts and dorsal root ganglia (DRG) were quickly removed, cleaned from the surrounding structures. Hearts were thoroughly washed in PBS to remove all blood. Hearts and DRG were quickly frozen in isopentane, cooled in liquid nitrogen, and stored at −80 °C until used for in situ hybridization (ISH), immunohistochemistry, and RT-qPCR analyses. Brains were quickly removed and cut along the midline. Left hemispheres were Golgi-stained or quickly frozen and used for Western blot analyses (see description below). Right hemispheres were fixed via immersion in 4 % paraformaldehyde (100 mM phosphate buffer, pH 7.4) for 48 hours, kept in 15–20 % sucrose for an additional 24 hours, and then frozen with dry ice.

For the analyses described below, animals were randomly selected from the experimental groups.

### Immunofluorescence procedures

#### Brain procedures

Hemispheres were cut with a freezing microtome into 30-μm-thick coronal sections that were serially collected and processed for immunohistochemistry procedures. One out of six sections from the hippocampal formation or somatosensory cortex were used for immunofluorescence for postsynaptic density protein 95 (PSD-95), vesicular glutamate transporter 1 (VGlut1), anti-allograft inflammatory factor 1 (AIF1), and glial fibrillary acidic protein (GFAP) following the protocol published in Refs. [33,34]. For the detection of Igk-TATk-CDKL5 protein, sections were incubated overnight at 4 °C with a primary anti-HA antibody and for 2 hours with an HRP-conjugated anti-rabbit secondary antibody. Detection was performed using the TSA Cyanine 3 Plus Fluorescein Evaluation Kits (PerkinElmer, Waltham, MA, USA). The sections were mounted with DAPI (40,6-diamidino-2-phenylindole)-Fluoromount-G (SouthernBiotech, Birmingham, AL, USA).

#### Heart procedures

Frozen hearts were cut with a cryostat (Histo-Line Laboratories, Pantigliate, MI, Italy) into 7 μm-thick sections. Sections were mounted on Superfrost™ slides and used for immunohistochemistry and in situ hybridization. Immunohistochemistry was performed on 7 μm-thick frozen sections, post-fixed by immersion in ice-cold methanol/ethanol (1:1), and permeabilized with 0.2 % Triton X-100 in PBS. Furthermore, 2 % BSA in PBS was used as a blocking reagent. Sections were incubated overnight with anti-γH2AX antibody washed with PBS, and subsequently incubated for 2 hours at room temperature with CY3-conjugated secondary antibodies. Nuclei were counterstained with DAPI.

#### DRG procedures

Frozen DRG were cut with a cryostat (Histo-Line Laboratories, Pantigliate, MI, Italy) into 15 μm-thick sections. Sections were mounted on SuperFrost® Plus slides, air-dried at room temperature, and stored at −80 °C until in situ hybridization.

The primary and secondary antibodies used in this study are listed in Supplementary Table 1.

### In Situ Hybridization (ISH)

Hemispheres were cut with a cryostat into 15-μm-thick coronal sections which were serially collected on super frost slides. ISH for CDKL5 mRNA on brain, heart, and DRG sections was performed using BaseScope™ R technology (Bio-Techne, Minneapolis, MN, USA) following the manufacturer's protocol, using a 1ZZ probe designed on the CDKL5 exon 4 [21]. For subsequent GFAP staining, immunofluorescence protocol was applied on slides right after ISH signal detection.

### Golgi staining

Hemispheres were Golgi-stained using the FD Rapid GolgiStain TM Kit (FD NeuroTechnologies, Columbia, MD, USA), as previously described [35]. Hemispheres were cut with a freezing microtome into 100-μm-thick coronal sections that were directly mounted onto gelatin-coated slides and were air dried at room temperature in the dark for an additional 2–3 days. After drying, sections were rinsed with distilled water and subsequently stained in the developing solution provided by the kit.

### Image acquisition and measurements

Fluorescence images were taken with an Eclipse TE 2000-S microscope equipped with a DS-Qi2 digital SLR camera (Nikon Instruments Inc., Tokyo, Japan). A light microscope (Leica Mycrosystems, Shinjuku City, Tokyo, Japan), equipped with a motorized stage and focus control system, and a color digital camera (Coolsnap-Pro, Media Cybernetics, Rockville, MD, USA) were used for neuronal tracing and to take bright field images. Measurements were carried out using the Image Pro Plus software (Media Cybernetics, Silver Spring, MD, USA).

#### Measurement of the dendritic tree

Dendritic trees were traced using custom-designed software for dendritic reconstruction (Immagini Computer, Milan, Italy), interfaced with Image Pro Plus as previously described [35]. Basal dendritic trees of Golgi-stained pyramidal neurons in layers II-III of the somatosensory cortex were traced live at a final magnification of 500×, by focusing on the depth of the section. The operator starts with branches emerging from the cell soma and, after having drawn the first parent branch, goes on with all daughter branches of the next order in a centrifugal direction. At the end of tracing, the program reconstructs the number and length of individual branches, the mean length of branches of each order, and the total dendritic length.

#### Dendritic spine number and morphology

In Golgi-stained sections, dendritic spines on the basal dendrites of pyramidal neurons in layers II–III of the somatosensory cortex were manually counted using a 100 × oil immersion objective lens (Leitz microscope and objective with 1.4 NA). For each mouse, 10–15 dendritic segments, each 10 μm in length, were analyzed, and spine density was expressed as number of spines per 10 μm. Based on their morphology, dendritic spines were classified into two groups reflecting their maturation state: immature and mature. The number of spines belonging to the 2 different groups (immature spines: filopodium-like, thin, and stubby-shaped; mature spines: mushroom- and cup-shaped) was counted and expressed as a percentage.

#### Quantification of PSD-95 and VGlut1 immunoreactive puncta

Images from the hippocampal CA1 layer and from the layers II-III of the somatosensory cortex were acquired using a LEICA TCS SL confocal microscope (LEITZ; Leica Microsystems, Wetzlar, Germany; objective 63x, NA 1.32; zoom factor = 8). Three to four sections per animal were analyzed and, for each section, three images from the regions of interest were captured and the density of individual puncta exhibiting PSD-95 or VGlut1 immunoreactivity was evaluated as previously described [36]; the number of immunoreactive puncta was expressed per μm^2^.

#### Morphometric microglial cell analysis

Starting from 20× magnification images of AIF-1-stained hippocampal and cortical slices, AIF-1 positive microglial cell body size was manually drawn using the Image Pro Plus measurement function and was expressed in μm^2^.

#### Cell density

The number of AIF-1-positive cells in the hippocampus and somatosensory cortex and the number of glial GFAP-positive cells in the hilus of the dentate gyrus was manually counted using the point tool of the Image Pro Plus software (Media Cybernetics, Silver Spring, MD, USA), and cell density was established as AIF-1-positive or GFAP-positive cells/mm^3^. The density of DAPI-positive nuclei in the CA1 layer was manually counted using the point tool of the Image Pro Plus software and expressed as cells/mm^3^.

#### Quantification of CDKL5 mRNA-positive cells

For the quantification of *CDKL5* mRNA positive cells, two images per section from the cortex, thalamus, and brain stem (n = 3–4 sections) were acquired using an Eclipse TE 2000-S microscope equipped with a DS-Qi2 digital SLR camera (Nikon Instruments Inc., Tokyo, Japan). All the positive cells present in the image were manually counted using the point tool of the Image Pro Plus software (Media Cybernetics, Silver Spring, MD, USA). The number of *CDKL*5 mRNA-positive cells was expressed as a percentage of the total number of cells, identified using DAPI staining.

#### Intensity-based analysis of CDKL5 mRNA expression in the hippocampus and cerebellum

Quantification of the levels of *CDKL5* mRNA expression in the CA1 and CA3 fields of the hippocampus and in the cerebellar cortex of mice was performed starting from 20× magnification ISH images using NIS-Elements AR software (Nikon Instruments Inc., Tokyo, Japan). An area was manually traced around each hippocampal or cerebellar region, and the average fluorescence intensity of the ISH staining, corresponding to the *CDKL5* mRNA signal, was quantified using software as the total intensity of all positive (bright) pixels within the defined area. Signal intensity was then normalized by subtracting the background intensity measured in each image. A total of six hippocampal or cerebellar slices were evaluated for each sample.

#### Quantification of γH2AX fluorescence intensity

Starting from 20× magnification images of ventricular slices processed for γH2AX immunohistochemistry, the average intensities of the γH2AX fluorescent signals were quantified by the software as the number of positive (bright) pixels within the selected area. A total of six heart slices were evaluated for each sample.

### Western blotting

For Western blot analysis cortical tissue samples were homogenized in RIPA buffer and quantified using the Bradford method, as previously described [34]. Equivalent amounts (50 μg) of protein were subjected to electrophoresis on a Bolt™ 4–12 % Bis-Tris Plus gel (Life Technologies Corporation, Carlsbad, CA, USA) and transferred to a Hybond ECL nitrocellulose membrane (GE Healthcare Bio- Science, Piscataway, NJ, USA). The primary and secondary antibodies used are listed in Supplementary Table 1. The densitometric analysis of digitized Western blot images was performed using Chemidoc XRS Imaging Systems and the Image Lab™ Software (Bio-Rad, Hercules, CA, USA); this software automatically highlights any saturated pixels in the Western blot images in red. Images acquired with exposition times that generated protein signals out of a linear range were not considered for the quantification.

### RNA isolation and RT-qPCR

RNA isolation and RT-qPCR were conducted on frozen hearts of GT-treated *Cdkl*5 +/−, and untreated *Cdkl*5 +/− and *Cdkl*5 +/+ mice. Total RNA was isolated using the TRI reagent method (Sigma-Aldrich, St. Louis, MO, USA), and cDNA synthesis was achieved with 5 μg of total RNA using an iScript™ advanced cDNA synthesis kit (Bio-Rad, Hercules, CA, USA) according to the manufacturer's instructions. Real-time PCR was performed using SsoAdvanced Universal SYBR Green Supermix (Bio-Rad, Hercules, CA, USA) in a CFX real-time PCR detection system (Bio-Rad). GAPDH (glyceraldehyde 3-phosphate dehydrogenase) was used as a reference gene for normalization in the qPCR. We used the primer pairs for *Cdkl5* and *Gapdh* described in Ref. [37]. Quantification was performed using the ΔΔCt method.

### Cytokine measurement

Circulating levels of the cytokines TNFα, IL-6, IL-1β, and IL-10 were measured in wild-type mice, *Cdkl5* +/− mice, and *Cdkl5* +/− mice treated with the AAVPHP.B_Igk-TATk-CDKL5 vector (+/− GT). Briefly, plasma was obtained by centrifugation of whole blood and used for a sandwich ELISA assay.

Ninety-six-well plates were coated with primary capture antibodies against TNFα (BioLegend Cat#510802, clone 6B8), IL-6 (BioLegend Cat#504502, clone MP5-20F3), IL-1β (BioLegend Cat#503502, clone B122), or IL-10 (BioLegend Cat#505002, clone JES5–16E3) at a final concentration of 2 μg/mL and incubated overnight at 4 °C. Plates were then blocked with PBS containing 10 % FBS at room temperature (RT) for 2 hours, followed by incubation at 4 °C overnight with either 50 μL of plasma or recombinant cytokine standards (BioLegend, San Diego, CA, USA), in technical duplicates. Following washes with 0.05 % Tween-20 in 1 × PBS (pH 7.4), plates were incubated for 1 hour at RT with biotinylated detection antibodies against TNFα (BioLegend Cat#506312, clone MP6-XT22), IL-6 (BioLegend Cat#504602, clone MP5-32C11), IL-1β (BioLegend Cat#515801, clone Poly5158), or IL-10 (BioLegend Cat#505004, clone ES5-16E3) at a final concentration of 1 μg/mL. After additional washing steps, plates were incubated with streptavidin-conjugated alkaline phosphatase for 30 minutes at RT in the dark. Finally, PNPP substrate was added, and colorimetric development was monitored. Absorbance was read at 405 nm using a Victor3 1420 Multilabel Counter (PerkinElmer, Waltham, MA, USA), and cytokine concentrations were quantified based on standard curves.

### Statistical analysis

Statistical analysis was performed using GraphPad Prism 8.0.1 (GraphPad Software, Boston, MA, USA). Statistical test and significance are reported in the Figure legends. Data are reported as means ± SEM or median [IQR], depending on whether the assumption of normality was met, with dots in each graph representing individual values. Outlier values were excluded according to the ROUT method (Q = 1 %). Gaussian and lognormal distribution of data sets was tested using D'Agostino-Pearson normality test or Shapiro-Wilk for low sample size. Lognormal datasets were transformed to their logarithmic values to approximate a normal distribution and meet the assumptions of parametric statistical tests. Equality of variances was tested using Brown-Forsythe test. Differences within datasets with normal distribution were analyzed for significance using Fisher's LSD or Tukey's test after One-Way ANOVA when the population had equal variances, or Unpaired *t*-test after Welch's ANOVA when unequal variance was present. Datasets with two independent variables were analyzed using Fisher's LSD after two-way ANOVA (repeated-measures ANOVA was applied when measures were paired within the same animal). Within-subject modeling was performed to assess overall time-dependent changes. Datasets with non-parametric distribution were analyzed using Uncorrected Dunn's test after Kruskal–Wallis. A probability level of p < 0.05 was considered to be statistically significant.

## Results

### Effects of gene therapy on behavior in heterozygous *Cdkl5* +/− female mice

**Fig. 1:**
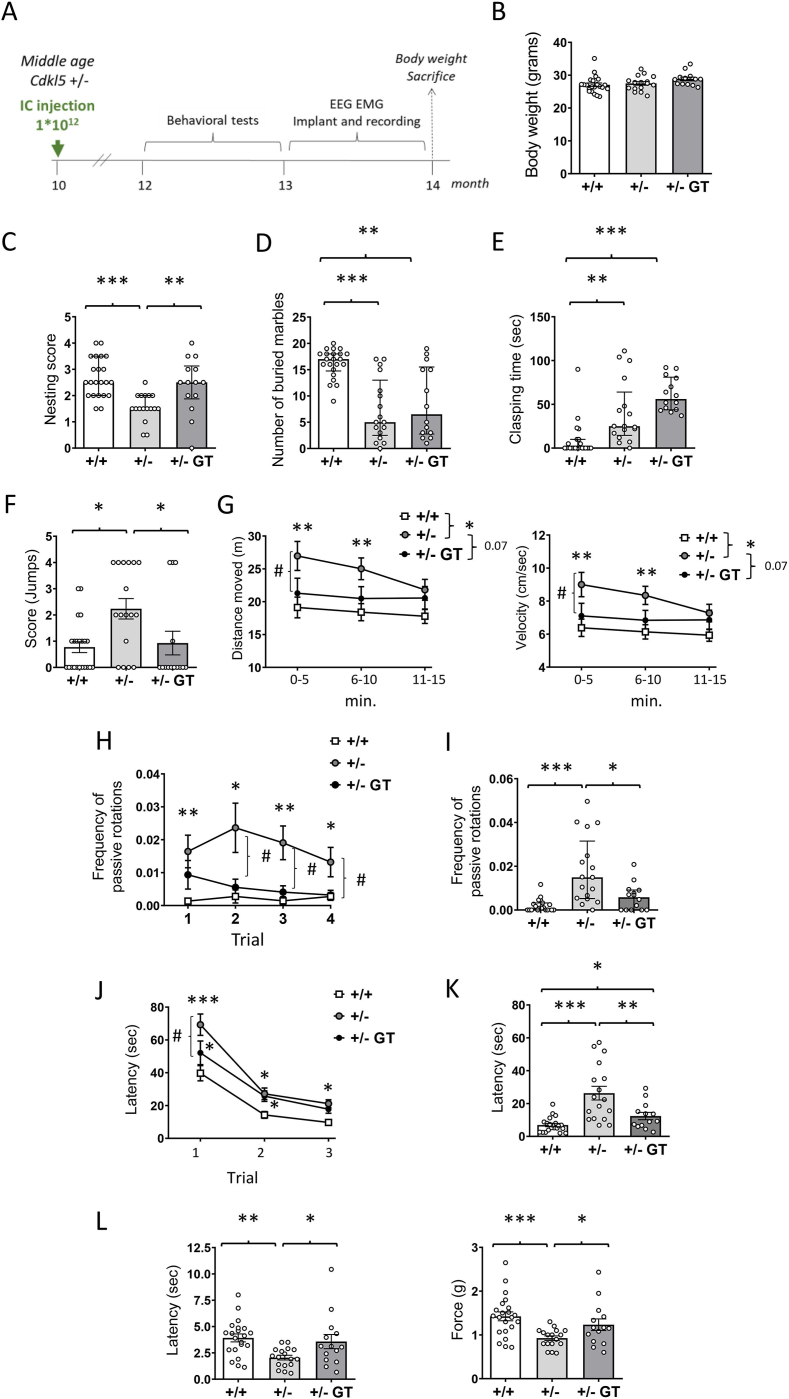
**Effect of Igk-TATk-CDKL5 gene therapy on behavior in middle-aged *Cdkl5*** ​+/− ​**mice.A**: Schematic representation of the experimental design. Ten-month-old *Cdkl5* ​+/− ​mice were systemically treated (intracarotid injection; IC) with the Igk-TATk-CDKL5 vector at a dose of 10^12^ vg/mouse. The effect of treatment on behavior was evaluated 2 months post-injection. Electroencephalographic (EEG) and electromyographic (EMG) recordings were performed 3 months post-injection. The body weight of the animals was recorded, and tissue samples were collected 4 months post-injection. **B**: Body weight (in grams) of wild-type (+/+; *n* ​= ​22) and *Cdkl5* ​(+/−; *n* ​= ​17) mice, and *Cdkl5* ​mice treated with the Igk-TATk-CDKL5 vector (+/− ​GT; *n* ​= ​14) according to the treatment schedule shown in A. **C,D**: Autistic-like features in treated *Cdkl5* ​+/− ​mice. Nest quality (**C**) and number of marbles buried (**D**) of wild-type (+/+; *n* ​= ​22) and *Cdkl5* ​(+/−; *n* ​= ​17) mice, and of *Cdkl5* ​mice 60 days after treatment with the Igk-TATk-CDKL5 vector (+/− ​GT*; n* ​= ​14). **E:** Total duration of hind-limb clasping behavior during a 2-min interval in mice as in B. **F**: Score of stereotypical jumps (repetitive beam breaks <1 ​s) in the corners of the open field arena during the 15-min trial in wild-type (+/+; *n* ​= ​22) and *Cdkl5* ​(+/−; *n* ​= ​17) mice, and in *Cdkl5* ​mice treated with the Igk-TATk-CDKL5 vector (+/− ​GT*; n* ​= ​14). **G:** Locomotor activity measured as total distance traveled (left graph) and average locomotion velocity (right graph) during a 15-min open field test in wild-type (+/+; *n* ​= ​22) and *Cdkl5* ​(+/−; *n* ​= ​12) mice, and in *Cdkl5* ​mice treated with the Igk-TATk-CDKL5 vector (+/− ​GT*; n* ​= ​13). **H,I:** Accelerating rotarod assay was performed in 4 trials with an intertrial interval of 1 ​h. Frequency of passive rotations (rotations in which the mouse does not perform any coordinated movement but is passively transported on the rotating apparatus) in every single trial (**H**) and overall frequency of passive rotations (**I**) of wild-type (+/+; *n* ​= ​19) and *Cdkl5* ​(+/−; *n* ​= ​17) mice, and of *Cdkl5* ​mice treated with the Igk-TATk-CDKL5 vector (+/− ​GT*; n* ​= ​13). **J,K:** Spatial learning assessed using the Barnes Maze in wild-type (+/+; *n* ​= ​21) and *Cdkl5* ​h(+/−; *n* ​= ​17) mice, and in *Cdkl5* ​mice treated with the Igk-TATk-CDKL5 vector (+/− ​GT*; n* ​= ​13). The graph in **J** shows the mean latency to find the target hole during the 3-day learning period. The graph in **K** shows the latency to find the target hole on the probe day (day 4). **L:** Behavioral assessment of pain sensitivity with von Frey filament test in wild-type (+/+; *n* ​= ​21) and *Cdkl5* ​(+/−; *n* ​= ​17) mice, and in *Cdkl5* ​mice treated with the Igk-TATk-CDKL5 vector (+/− ​GT*; n* ​= ​14). Mechanical allodynia was quantified by measuring the hind paw withdrawal latency to von Frey filament stimulation (graph on the left) and the force in grams at which the paw withdrawal threshold was reached (graph on the right). Values are presented as means ​± ​SEM in (B, G, H, and J-L) or median [IQR] in (C, D, E, F and I). ∗*p* ​< ​0.05; ∗∗*p* ​< ​0.01; ∗∗∗*p* ​< ​0.001 as compared to *Cdkl5* +/+ condition; #p ​< ​0.05 as compared to the *Cdkl5* ​+/− ​condition. Tukey's test after one-way ANOVA for datasets in (B). Fisher's LSD test after one-way ANOVA for data set in (K–L) and after two-way RM ANOVA for data set in (G, H and J). Uncorrected Dunn's test after Kruskal-Wallis for data set in (C–F, and I). Within-subject modeling for data sets in (G).

To assess the effect of gene therapy in the presence of mosaic neuronal loss of CDKL5, middle-aged (10-month-old) *Cdkl5 +/−* mice were systemically administered with AAVPHP.B_Igk-TATk-CDKL5 vector at a dose of 10^12^ vg/mouse via intracarotid injection (Fig. 1A). Given that heterozygous *Cdkl5* +/− female mice develop spontaneous seizures with aging, closely recapitulating the epileptic phenotype observed in CDD patients [16,17], we specifically selected mice older than 10 months for this study. Untreated age-matched *Cdkl5* +/− and wild-type (+/+) mice were used as controls. To assess the impact of the treatment on behavior, mice underwent a battery of tests two months after administration, evaluating spontaneous home-cage behaviors, locomotor activity and coordination, hippocampal-dependent memory, and nociception. Following behavioral testing, mice were implanted with electrodes to acquire fronto-parietal differential electroencephalographic (EEG) and electromyographic (EMG) recordings. At four months post-injection, animals were weighed prior to sacrifice (Fig. 1A). No difference in body weight was observed in gene therapy (GT)-treated *Cdkl5* +/− mice compared to age-matched untreated mice (Fig. 1B), suggesting that the treatment did not adversely affect general animal health.

#### Innate home cage behaviors

Loss of CDKL5 function in *Cdkl5* +/− female mice is associated with impaired nesting and reduced marble burying compared to wild-type mice (Fig. 1C and D) [15]. GT-treated *Cdkl5* +/− mice showed a significant improvement in nest building compared to the untreated *Cdkl5* +/− mice, with nest building ability restored to the level of wild types (Fig. 1C). In contrast, no improvement was observed in the number of marbles buried in GT-treated *Cdkl5* +/− mice compared to the untreated *Cdkl5* +/− group (Fig. 1D).

#### Stereotypic movements

Stereotypic movements characterize *Cdkl5* +/− mice [15] and CDD patients [6]. In order to examine the effect of gene therapy on motor stereotypies, mice were tested for hindlimb clasping. Despite a clear difference being observed between wild-type (+/+) and *Cdkl5* +/− female mice (Fig. 1E), no improvement was detected in GT-treated animals compared to untreated *Cdkl5* +/− mice (Fig. 1E). As an additional stereotypic behavior, *Cdkl5* +/− mice showed an increased number of repetitive jumps, displayed as intense clusters of jumps or a smaller number of jumps but in multiple consecutive sessions, when placed in an open field arena (Fig. 1F). GT treatment efficiently decreased stereotypic jumps in treated animals compared to *Cdkl5* +/− female mice (Fig. 1F).

#### Motor functions

To assess whether motor impairment was reduced by GT treatment, we evaluated locomotor activity using an open field arena. The increased locomotor activity (longer distance traveled with a higher average speed) that characterizes *Cdkl5* +/− female mice was clearly improved by the GT treatment (Fig. 1G). Additionally, *Cdkl5* +/− female mice showed a deficit in locomotor habituation during the 15 min of the open field testing session, with a clear trend toward improvement (p = 0.07) following GT treatment. To further evaluate locomotor activity, we assessed motor functions on an accelerating rotarod assay (Fig. 1H and I). As previously shown [15], *Cdkl5* +/− mice showed a significantly higher frequency of passive rotations compared to wild-type mice (number of passive rotations/sec) when placed on a rotating rod (Fig. 1H and I). In contrast, GT-treated *Cdkl5* +/− mice exhibited improved motor coordination, as indicated by a reduced frequency of passive rotations, reaching levels comparable to those observed in wild-type mice (Fig. 1H and I).

#### Spatial learning and memory

To examine the effect of GT on the cognitive impairment characterizing *Cdkl5* +/− mice [15,21], hippocampus-dependent learning and memory were evaluated using the Barnes maze. Interestingly, an improvement was observed in spatial orientation ability during the first day of the trial but not in the consecutive days in GT-treated vs. untreated *Cdkl5* +/− mice (Fig. 1J). It is relevant to note that a clear memory improvement, seen in the decreased latency to find the hole, was present in GT-treated *Cdkl5* +/− mice on the last day during the probe test (Fig. 1K).

#### Hyperalgesia

Alteration in pain perception, in particular, enhanced sensitivity, is a common characteristic in CDD patients [38]. Behavioral assessment of pain sensitivity with noxious mechanical stimulation *(von Frey*; paw withdrawal latency and actual force at the time of paw withdrawal) showed significantly increased sensitivity in *Cdkl5* +/− mice (Fig. 1L). Interestingly, GT treatment restored pain perception in *Cdkl5* +/− mice, with paw withdrawal latency and actual force coming close to those of wild types (Fig. 1L).

### Effects of gene therapy on seizure activity and wake-sleep behavior in heterozygous *Cdkl5* +/− female mice

**Fig. 2:**
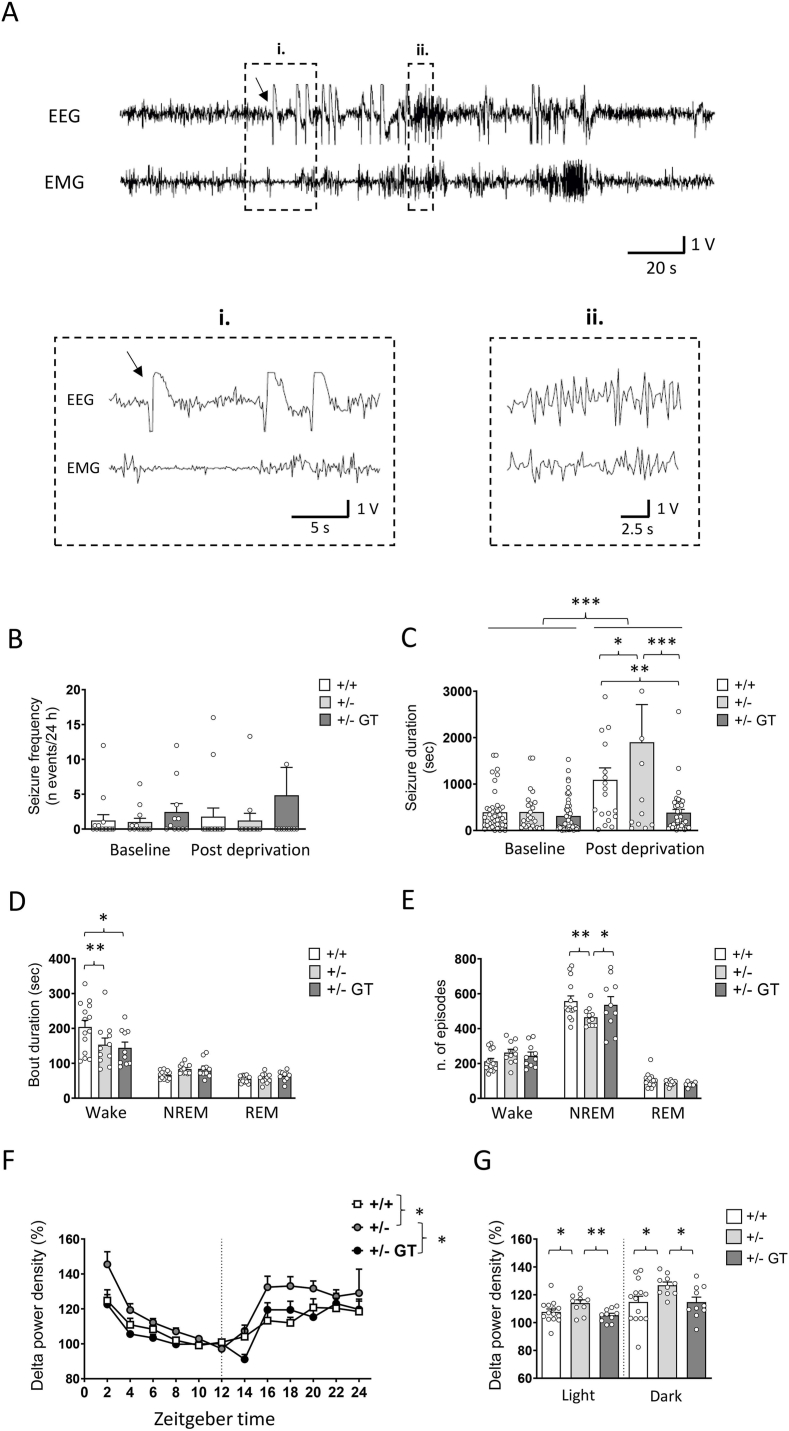
**Effect of Igk-TATk-CDKL5 gene therapy on spontaneous epileptic spasms, sleep architecture, and EEG delta power in middle-aged *Cdkl5*** ​+/− ​**mice. A:** Representative seizure captured by electroencephalography (EEG) and electromyography (EMG) and magnifications corresponding to its onset (i; marked with the arrow) and evolution (ii). **B:** Frequency of epileptic seizures in wild-type (+/+; *n* ​= ​15) and *Cdkl5* ​(+/−; *n* ​= ​13) mice, and *Cdkl5* ​mice treated with the Igk-TATk-CDKL5 vector (+/− ​GT*; n* ​= ​11) evaluated over 48 ​h of recording condition (Baseline) and for 18 ​h in sleep post-deprivation phase (Post deprivation). **C:** Seizure duration of mice as in B. **D**: Average duration of Wake, NREM, and REM episodes in wild-type (+/+; n ​= ​14) and *Cdkl5* ​(+/−; n ​= ​11) mice, and *Cdkl5* ​mice treated with the Igk-TATk-CDKL5 vector (+/− ​GT; n ​= ​10). **E**: Number of Wake, NREM, and REM sleep episodes over 48 ​h of baseline recording in the same animals as in D. **F:** Distribution of EEG delta (1.0–4.0 ​Hz) power density (power/minute) recorded as a function of the light/dark cycle (Zeitgeber time) in EEG analysis of wild-type (+/+; *n* ​= ​15) and *Cdkl5*(+/−; *n* ​= ​13) mice, and of *Cdkl5* ​mice treated with the Igk-TATk-CDKL5 vector (+/− ​GT*; n* ​= ​11). Data are given as percentages of the lowest levels reached in baseline (8–12 ​h). **G:** Mean EEG delta power density during the light or dark periods in mice as in D. Values are presented as means ​± ​SEM. ∗*p* ​< ​0.05; ∗∗*p* ​< ​0.01; ∗∗∗*p* ​< ​0.001 as compared to the *Cdkl5* +/+ condition. Fisher's LSD test after two-way RM ANOVA for datasets in (B, C) or without repeated measure for dataset in (D, E). Fisher's LSD after one-way ANOVA for data set in (G-light and G-dark). Whitin-subject modeling for data set in (F).

Simultaneous recordings of EEG and EMG activity were performed on mice that were undisturbed and freely behaving in their own cages continuously for 48 h in normal (baseline) conditions and for 18 h in sleep post-deprivation phase. Videos of the mice and EEG traces were analyzed to identify epileptic seizures. As previously reported in a CDD mouse model carrying a *Cdkl5* exon 6 deletion [16,17], we noted, albeit very rarely, the spontaneous occurrence of myoclonic and tonic-clonic seizures in middle-age heterozygous female mice with the *Cdkl5* exon 4 deletion (Supplementary Videos 1 and 2). More frequently, we observed spasms associated to interictal activity as previously shown [16,17], while epileptic seizures (Fig. 2A) were mainly represented by brief stiffening of the tail, resembling Straub tail [39], or facial automatisms, and occurred in both the light and dark phases. We were unable to identify differences in seizure frequency (Fig. 2B), duration (Fig. 2C) and severity (data not shown) between wild-type and *Cdkl5* +/− mice during the 48 h of recording in baseline conditions, showing great heterogeneity in spontaneous seizures as expected [17]. Importantly, treatment with GT did not worsen the epileptic phenotype in *Cdkl5* +/− mice (Fig. 2B and C). Across our cohort of mice, 5 out of the 17 wild-type (29.41 %), 5 out of the 13 *Cdkl5* +/− mice (38.46 %) and 6 out of 11 GT-treated *Cdkl5* +/− mice (54.54 %) showed epileptic seizures (p > 0.05, Chi-Square Test). Most events were observed during wakefulness or at the transition from wakefulness to NREM sleep across all experimental groups. When mice were subjected to a stress condition, such as the sleep deprivation, we observed that while seizure frequency was not different than in basal conditions in wild-type, *Cdkl5* +/− and GT-treated *Cdkl5* +/− mice (Fig. 2B), a relevant increase in seizure duration between baseline and post-deprivation phases was observed in wild-type and *Cdkl5* +/− mice; interestingly, the GT treatment was able to shorten the seizure duration in the post-deprivation phase and bring it to similar values observed in baseline conditions (Fig. 2C), suggesting that the observed seizure worsening under stress conditions in *Cdkl5* +/− mice was, at least in part, due to the lack of CDKL5.

Supplementary video related to this chapter can be found at https://doi.org/10.1016/j.neurot.2025.e00727

The following is/are the supplementary data related to this chapter:

VideoS1 3 VideoS1

VideoS2 4 VideoS2

Regarding wake-sleep behavior, the absence of CDKL5 did not significantly affect the percentage of recording time spent in wakefulness, NREM sleep, or REM sleep during the dark phase (Supplementary Table 2). In contrast, *Cdkl5* +/− mice showed a significant reduction in the percentage of time spent awake during the light phase compared to wild-type mice, accompanied by a corresponding increase in the time spent in NREM sleep (Supplementary Table 2). No significant differences were observed in the percentage of time spent in REM sleep between *Cdkl5* +/− and wild-type mice during either the light or dark phase (Supplementary Table 2). Following gene therapy, *Cdkl5* +/− mice showed no difference in the percentage of wakefulness, NREM sleep, or REM sleep in comparison to untreated *Cdkl5* +/− mice (Supplementary Table 2). The average duration (Fig. 2D) of wake-sleep episodes did not significantly differ between groups, except for the duration of wakefulness episodes, which was significantly longer in wild-type mice compared to both *Cdkl5* +/− and *Cdkl5* +/− GT mice (Fig. 2D). This justifies the significant reduction in the percentage of time spent awake during the light phase in *Cdkl5* +/− mice. Of note, the number of NREM episodes (Fig. 2E) was lower in *Cdkl5* +/− mice, and gene therapy treatment effectively rescued the number of episodes to levels comparable to those observed in wild-type mice (Fig. 2E). One-way ANOVA revealed no significant differences in the sleep fragmentation index among the experimental groups (Supplementary Table 2).

Power spectral densities across all frequency bands, analyzed over a 24 h period, showed that *Cdkl5* +/− mice had higher delta power density in comparison with wild-type mice (Fig. 2F), in both light and dark phases (Fig. 2F and G). Notably, GT treatment restored the delta spectral densities in *Cdkl5* +/− mice to those of the control condition (Fig. 2F and G).

### Effect of gene therapy on dendritic and synaptic organization in heterozygous *Cdkl5* +/− female mice

**Fig. 3:**
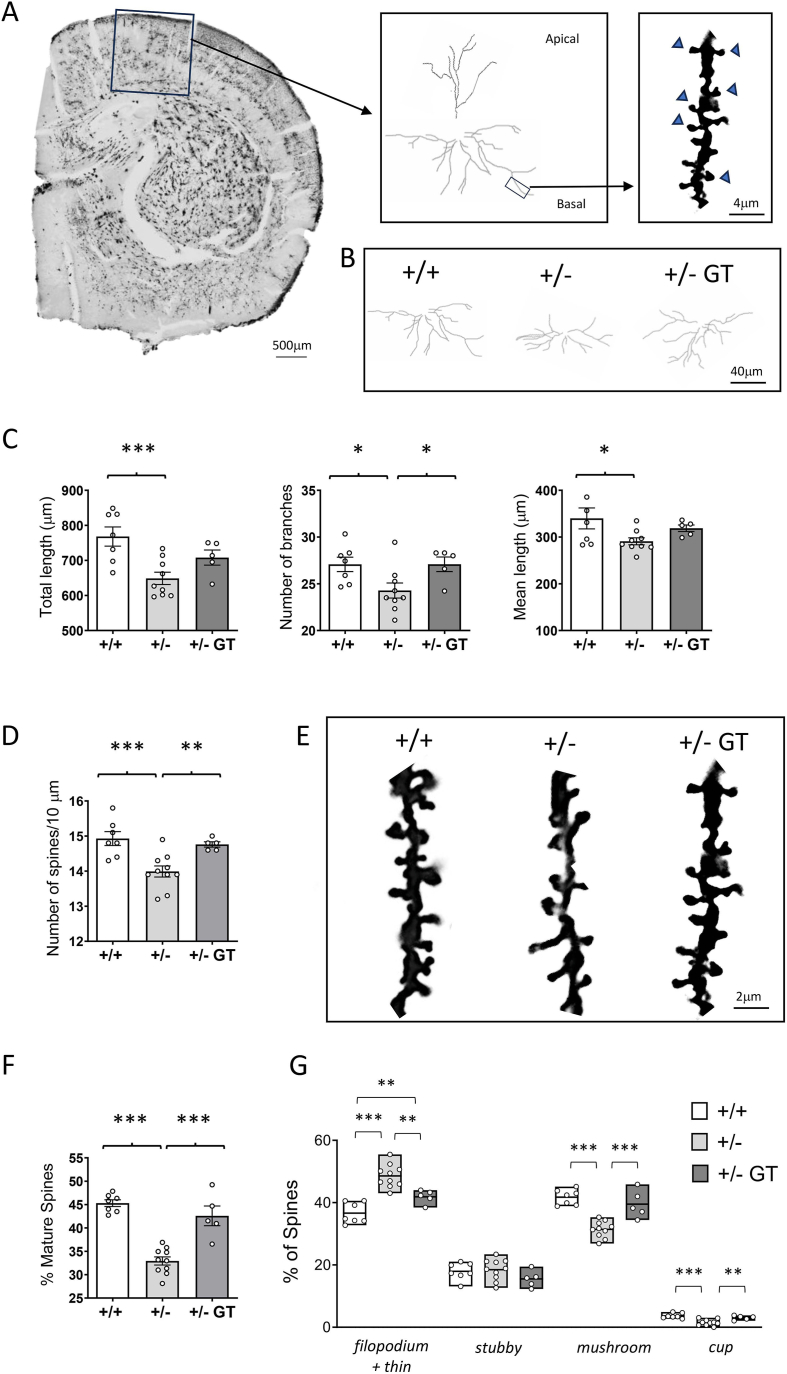
**Effect of Igk-TATk-CDKL5 gene therapy on dendritic morphology in middle-aged *Cdkl5*** ​+/− **mice. A:** Representative image of a Golgi-stained section (panel on the left; scale bar ​= ​500 ​μm), showing the portion of the cortical region of a wild-type mouse in which the dendritic arbor of pyramidal neurons was traced (areas enclosed in the square). The central panel shows an example of the reconstructed apical and basal dendritic tree of a Golgi-stained cortical pyramidal neuron. The right panel presents a higher-magnification view of a basal dendritic branch (outlined in the square) from a Golgi-stained pyramidal neuron, highlighting dendritic spines, with blue arrowheads indicating mature spines. Scale bar ​= ​4 ​μm. **B:** Examples of the reconstructed basal dendritic tree of a Golgi-stained cortical pyramidal neuron of one wild-type (+/+; on the left), one *Cdkl5* (+/−; in the middle), and one *Cdkl5* ​mouse treated with the Igk-TATk-CDKL5 vector (+/− ​GT; on the right). Scale bar ​= ​40 ​μm. **C:** Comparison between the total dendritic length (on the left), the number of branches (in the middle), and the mean length (on the right) of basal dendritic branches of Golgi-stained pyramidal neurons in the somatosensory cortex of wild-type mice (+/+; *n* ​= ​7), *Cdkl5* ​mice (+/−; *n* ​= ​9), and *Cdkl5* ​hmice treated with the Igk-TATk-CDKL5 vector (+/− ​GT*; n* ​= ​5). **D:** Dendritic spine density of cortical pyramidal neurons from wild-type mice (+/+; *n* ​= ​7) and *Cdkl5* ​(+/−; *n* ​= ​10) mice, and from *Cdkl5* ​mice treated with the Igk-TATk-CDKL5 vector (+/− ​GT*; n* ​= ​5). **E:** Representative images of basal dendrites of Golgi-stained cortical pyramidal neurons, illustrating mature and immature spines of one animal from each experimental group. Scale bar ​= ​2 ​μm. **F:** Percentage of mature dendritic spines in relation to the total number of protrusions in cortical pyramidal neurons of mice as in D. **G:** Percentage of immature and mature dendritic spines of each morphological class in relation to the total number of protrusions in cortical pyramidal neurons of mice as in D. Values are represented as means ​± ​SEM. ∗*p* ​< ​0.05, ∗∗*p* ​< ​0.01, ∗∗∗*p* ​< ​0.001. Fisher's LSD test after one-way ANOVA in for data sets in (C,D and F); Fisher's LSD test after two-way ANOVA for data set in (G).

Behavioral defects in heterozygous *Cdkl5* +/− female mice are associated with brain morphological defects, including decreased dendritic branching in hippocampal and cortical neurons, reduced dendritic spine density and organization, and reduced connectivity [15]. In order to establish the effect of gene therapy on these brain morphological defects, we analyzed basal dendritic development and spine density/morphology in Golgi-stained pyramidal neurons in layer II/III of the somatosensory cortex (Fig. 3A and B). *Cdkl5* +/− mice exhibit impaired dendritic arborization compared to wild-type mice, with reduced dendritic length, number of branches, and mean length of segments (Fig. 3C). It is important to note that due to the nature of the Golgi method, simultaneous labeling for CDKL5 expression was not feasible. Therefore, we could not directly distinguish between Cdkl5-positive and Cdkl5-negative neurons during the analysis. Given the mosaic expression of CDKL5 in heterozygous *Cdkl5* female mice, the data reflect a mixture of approximately 50% Cdkl5-deficient neurons and 50% wild-type neurons. Treatment with GT ameliorated the cortical neuronal network defects by improving neuronal length and rescuing branch complexity (Fig. 3C).

Spine density reduction is a typical feature of the dendritic pathology that affects the brain of *Cdkl5* +/− mice [15]. In addition, *Cdkl5* +/− mice exhibit a deficit in dendritic spine structure and stabilization [15]. We observed a reduction in spine density (Fig. 3D and E), along with defects in spine maturation, characterized by fewer mature (mushroom-like and cup-shaped) spines (Fig. 3F and G) and an increase in immature filopodia-like spines (Fig. 3G). Spine density (Fig. 3D) and the percentage of mature spines (Fig. 3F and G) were restored in *Cdkl5* +/− mice following gene therapy treatment.

**Fig. 4:**
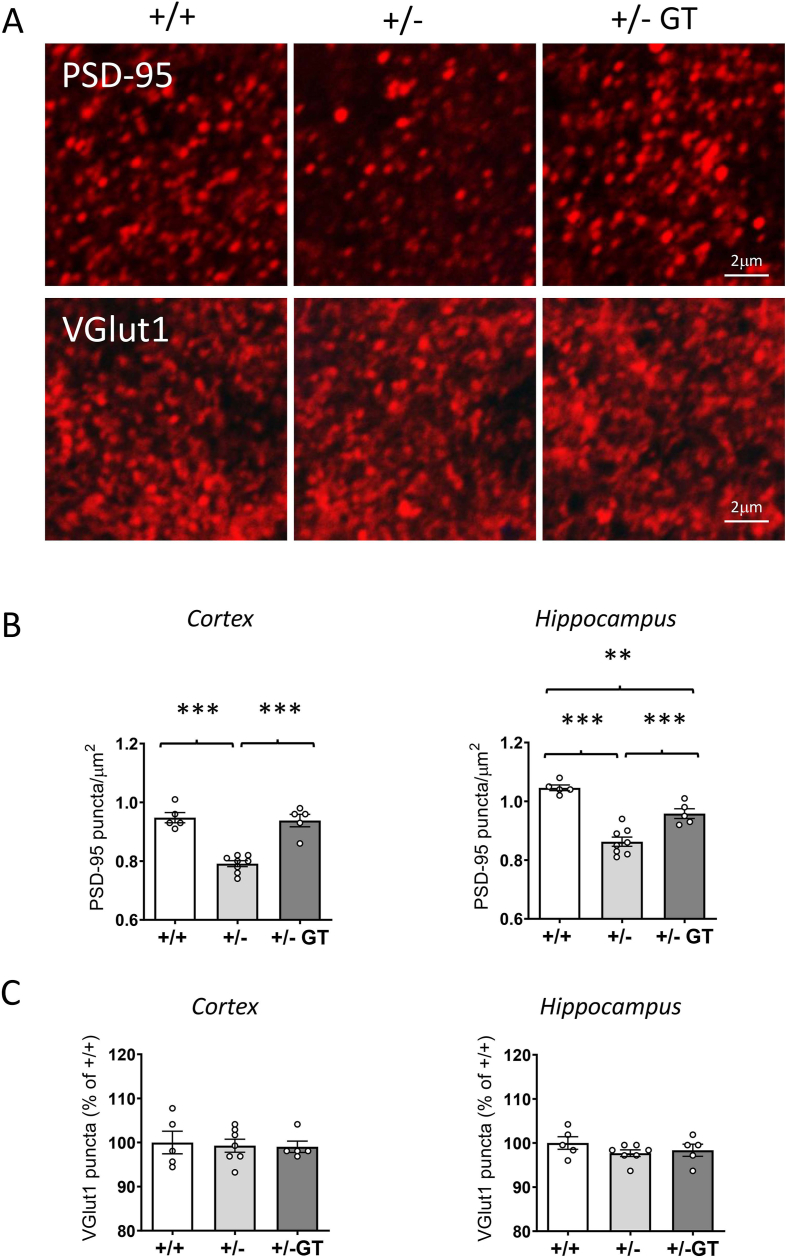
**Effect of Igk-TATk-CDKL5 gene therapy on connectivity in middle-aged *Cdkl5*** ​+/− ​**mice**. **A:** Representative confocal images of cortical sections processed for PSD-95 (upper images) and VGlut1 (lower images) immunohistochemistry from a wild-type (+/+) and a *Cdkl5* ​ (+/−) mouse, and from a *Cdkl5* ​mouse treated with the Igk-TATk-CDKL5 vector (+/− ​GT). Scale bar ​= ​2 ​μm. **B:** Number of fluorescent puncta per μm^2^, exhibiting PSD-95 immunoreactivity in the somatosensory cortex (histogram on the left) and in the CA1 layer of the hippocampus (histogram on the right) of wild-type (+/+; *n* ​= ​5) and *Cdkl5* ​(+/−; *n* ​= ​5) mice, and of *Cdkl5* ​mice treated with the Igk-TATk-CDKL5 vector (+/− ​GT*; n* ​= ​5). **C:** Number of fluorescent puncta per μm^2^, exhibiting vesicular glutamate transporter 1 (VGlut1) immunoreactivity in the somatosensory cortex (histogram on the left) and in the CA1 layer of the hippocampus (histogram on the right) of mice as in B. Data are expressed as percentages of the wild-type condition. Values are represented as means ​± ​SEM. ∗∗*p* ​< ​0.01, ∗∗∗*p* ​< ​0.001. Fisher's LSD test after one-way ANOVA.

Next, we investigated excitatory synapse organization in both the cortex and hippocampus of *Cdkl5* +/− mice by analyzing the number of the postsynaptic density protein 95 (PSD-95) clusters and the vesicular glutamate transporter 1 (VGlut1), which is a marker of glutamatergic axon terminals [40]. Evaluation of PSD-95 immunoreactivity showed that *Cdkl5* +/− mice displayed a strong reduction in the number of PSD-95-positive puncta in both the cortex and hippocampus (Fig. 4A and B), which is consistent with the reduced number of mature spines. On the contrary, no difference in the number of excitatory terminals (VGlut1-positive puncta, Fig. 4A) was observed in the layer II/III of the somatosensory cortex and in the stratum radiatum of CA1 field in *Cdkl5* +/− mice in comparison to wild-type mice (Fig. 4C). The number of PSD-95 puncta was restored in the cortex and improved in the hippocampus of GT-treated *Cdkl5* +/− mice (Fig. 4A and B), while no change in the number of VGlut1 puncta was observed (Fig. 4A and C).

### Effect of gene therapy on microglial activation and hippocampal neuronal survival in heterozygous *Cdkl5* +/− female mice

**Fig. 5:**
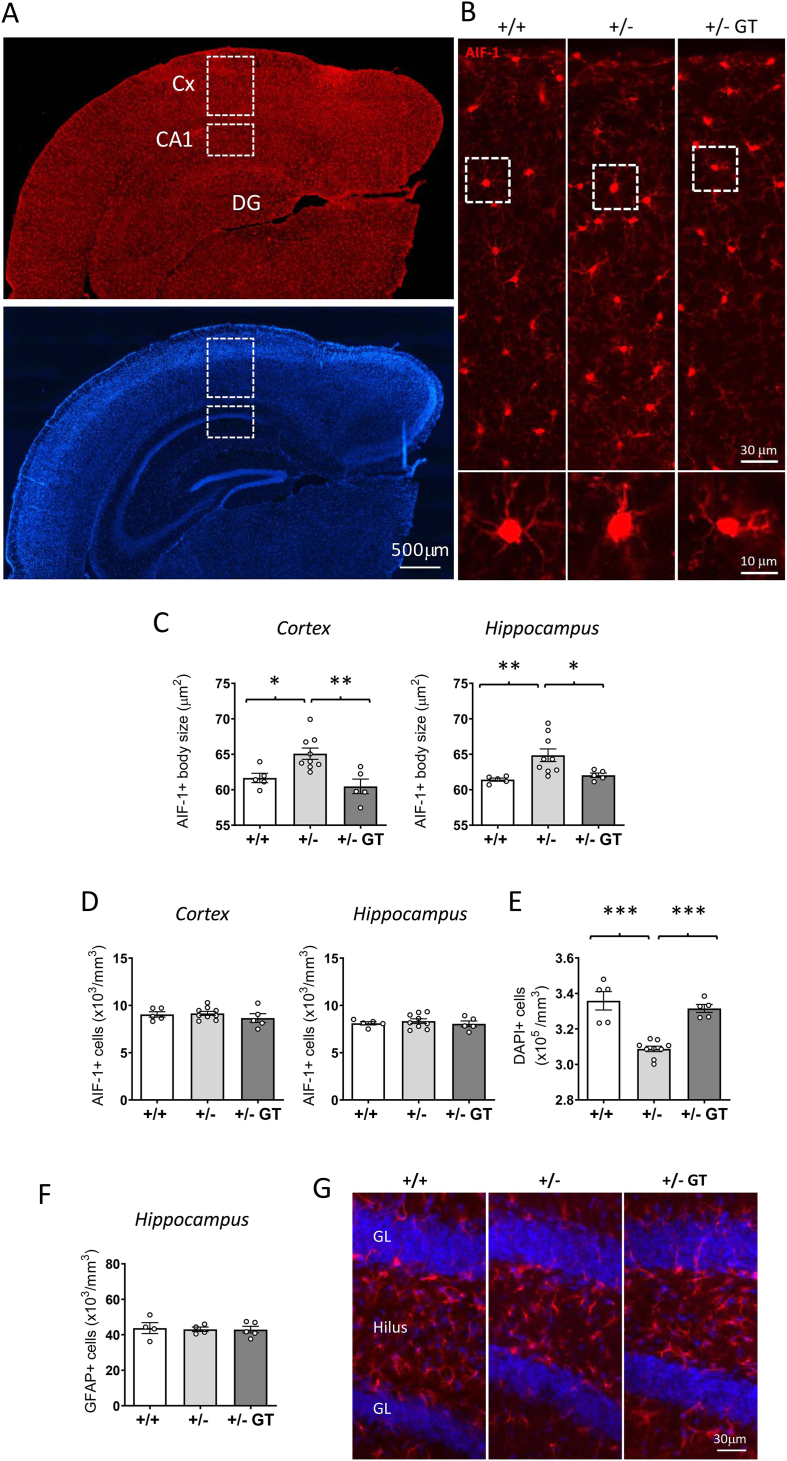
**Effect of Igk-TATk-CDKL5 gene therapy on microglia activation, hippocampal neuronal survival, and glia cell number in the brain of middle-aged *Cdkl5*** ​+/− ​**mice. A:** Representative fluorescence image of a wild-type mouse brain section processed for AIF-1 immunohistochemistry (upper panel), showing the portion of the cortex and of the hippocampal region in which the number of microglial cells were evaluated (areas enclosed in the dashed squares). Nuclei were counterstained with DAPI (lower panel). Scale bar ​= ​500 ​μm. **B:** Representative fluorescence images of cortical sections processed for AIF-1 immunohistochemistry in a wild-type (+/+) and a *Cdkl5* ​(+/−) mouse, and a *Cdkl5* ​mouse treated with the Igk-TATk-CDKL5 vector (+/− GT). The dotted boxes in the upper panels indicate the regions containing microglial cells, which are shown in magnification in the lower panels. Scale bars represent 30 ​μm for low-magnification images and 10 ​μm for high-magnification images. **C**: Mean microglia cell body size in the somatosensory cortex (upper histogram) and in the hippocampal region (lower histogram) of wild-type (+/+; *n* ​= ​5) and *Cdkl5* ​(+/−; *n* ​= ​9) mice, and of *Cdkl5* ​mice treated with the Igk-TATk-CDKL5 vector (+/− ​GT*; n* ​= ​5). **D:** Quantification of AIF-1-positive cells in the somatosensory cortex (histogram on the left) and in the hippocampal region (histogram on the right) of mice as in B. **E:** Quantification of DAPI-positive cells in the CA1 field of the hippocampus of mice as in B. **F:** Quantification of glial fibrillary acidic protein (GFAP)-positive cells in the hilus of the dentate gyrus of wild-type (+/+; *n* ​= ​4) and *Cdkl5* ​(+/−; *n* ​= ​4) mice, and of *Cdkl5* ​mice treated with Igk-TATk-CDKL5 vector (+/− ​GT*; n* ​= ​5). **G:** Representative fluorescent images of brain sections immunostained for GFAP (red) and counterstained with DAPI (blue) from a wild-type (+/+) and a *Cdkl5* ​(+/−) mouse, and from a *Cdkl5* ​mouse treated with the Igk-TATk-CDKL5 vector (+/− ​GT). Scale bar ​= ​30 ​μm. Values in B-E are represented as means ​± ​SEM. ∗*p* ​< ​0.05, ∗∗*p* ​< ​0.01, ∗∗∗*p* ​< ​0.001. Fisher's LSD test after one-way ANOVA. Abbreviations: Cx ​= ​somatosensory cortex, CA1 ​= ​hippocampal CA1 field, DG ​= ​dentate gyrus, GL ​= ​granule cell layer of the dentate gyrus.

*Cdkl5* +/− female mice are characterized by increased microglial activation [34], and decreased survival of hippocampal neurons [41]. The enlarged body size of microglial cells (AIF-1-positive cells) in *Cdkl5* +/− mice vs. wild-type mice was reverted in both the cortex and hippocampus of GT-treated *Cdkl5* +/− mice (Fig. 5A–C), suggesting a reduction in microglial activation. No change in terms of microglial cell number was observed in GT-treated *Cdkl5* +/− mice compared with untreated mice (Fig. 5D), indicating that GT treatment did not cause neuroinflammation. To assess the efficacy of GT treatment on neuronal survival, we evaluated the density of DAPI-positive nuclei in the CA1 layer of the hippocampus of *Cdkl5* +/− mice. Female *Cdkl5* +/− mice showed a reduced number of pyramidal neurons in the CA1 layer compared with wild-type mice (Fig. 5E), while GT treatment restored neuronal number in *Cdkl5* +/− mice (Fig. 5E). In order to evaluate whether CDKL5 expression in glial cells, that do not express CDKL5, has a harmful effect, we assessed the number of GFAP-positive cells in the hippocampus. No difference in glial cell number was observed among *Cdkl5* +/+, *Cdkl5* +/−, and GT-treated *Cdkl5* +/− mice (Fig. 5F and G).

### Efficacy of gene therapy on CDKL5 replacement in the brain of heterozygous *Cdkl5* +/− female mice

**Fig. 6:**
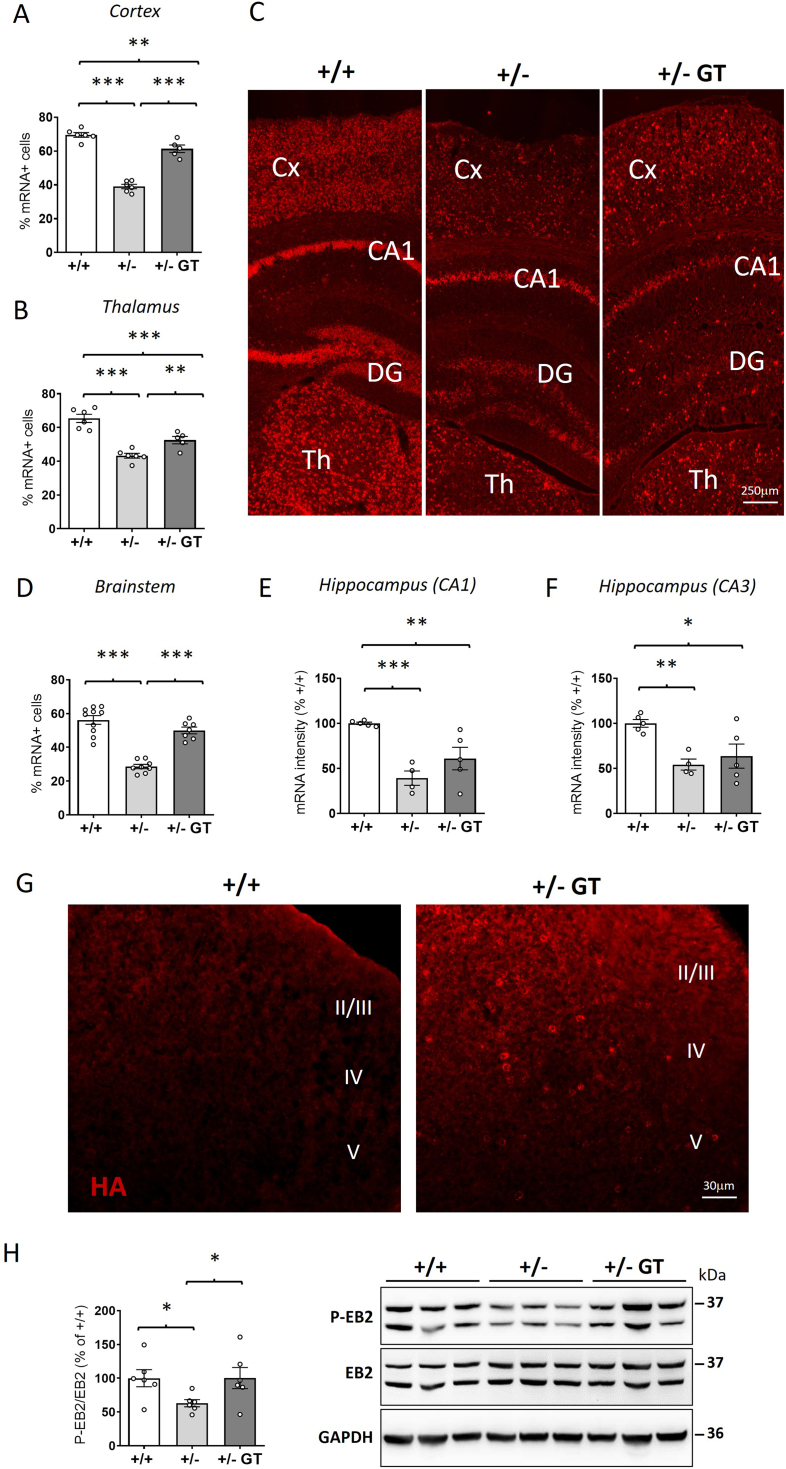
**TATk-CDKL5 expression in the brain of middle-aged *Cdkl5*** ​+/− ​**mice. A,B:** Quantification of the levels of *CDKL5* mRNA expression in the cortex (A) and thalamus (B) of wild-type (+/+; *n* ​= ​6) and *Cdkl5* ​(+/−; *n* ​= ​6) mice, and *Cdkl5* ​mice treated with the gene therapy the Igk-TATk-CDKL5 vector (+/− ​GT; *n* ​= ​5). Histograms show the percentage of *CDKL5* mRNA expressing cells. **C:** Representative images of brain sections with fluorescence in situ hybridization (ISH) for *CDKL5* mRNA (red) of a wild-type (+/+) and a *Cdkl5* mouse (+/−) mouse, and of a *Cdkl5* ​mouse treated with the Igk-TATk-CDKL5 vector (+/− ​GT). Scale bar ​= ​250 ​μm. **D:** Quantification of the levels of *CDKL5* mRNA expression in the brainstem of wild-type (+/+; *n* ​= ​6) and *Cdkl5* ​(+/−; *n* ​= ​6) mice, and of *Cdkl5* ​mice treated with the Igk-TATk-CDKL5 vector (+/− ​GT; *n* ​= ​5). Data are given as a percentage of *CDKL5* mRNA expressing cells. **E,F:** Quantification of the levels of *CDKL5* mRNA expression in the CA1 (E) and CA3 (F) fields of the hippocampus of mice as in A. The *CDKL5* mRNA expression was evaluated as mean intensity of *CDKL5* mRNA staining per area. Data are expressed as percentages of the wild-type condition. **G:** Representative fluorescence images of cortical sections, processed for anti HA immunostaining, showing TATk-CDKL5 protein expression in the cortex of a wild-type (+/+) and of a *Cdkl5* ​(+/−) mouse treated with the Igk-TATk-CDKL5 vector (+/− ​GT). Scale bar ​= ​30 ​μm. **H:** Western blot analysis of phospho-EB2 and total EB2 in cortical protein extracts from wild-type (+/+; *n* ​= ​6) and *Cdkl5* ​(+/−; *n* ​= ​6) mice, and from *Cdkl5* ​mice treated with the Igk-TATk-CDKL5 vector (+/− ​GT*; n* ​= ​6). Histogram on the left shows protein levels of phosphorylated EB2 normalized to the respective total form in cortex homogenates from mice; data are expressed as a percentage of the wild-type condition. Example of immunoblots from three animals of each experimental group on the right. Values in A,B, D-F and H represent means ​± ​SEM. ∗*p* ​< ​0.05, ∗∗*p* ​< ​0.01, ∗∗∗*p* ​< ​0.001. Fisher's LSD test after one-way ANOVA. Abbreviations: Cx ​= ​somatosensory cortex, CA1 ​= ​hippocampal CA1 field, DG ​= ​dentate gyrus, TH ​= ​thalamus, II/III, IV and V = Cortical layers.

By counting the number of *CDKL5* mRNA-positive cells in brain slices processed for in situ hybridization (ISH), we found that in the cortex (Fig. 6A and C, and Supplementary Fig. 1), thalamus (Fig. 6B and C, and Supplementary Fig. 1), and brain stem (Fig. 6D and Supplementary Fig. 2A) of wild-type mice approximately 60–70% of cells express CDKL5ì. As expected, this number was reduced to approximately half (30–40%) in *Cdkl5* +/− mice (Fig. 6A–D, Supplementary Fig. 1, and Supplementary Fig. 2A). In high cellular density regions such as hippocampal layers (CA1 and CA3), *CDKL5* mRNA expression was quantified as fluorescence intensity. Even in these regions, *CDKL5* mRNA levels in heterozygous *Cdkl5* +/− female mice were approximately half of those in wild-type mice (Fig. 6E and F, and Supplementary Fig. 1). As previously reported [21], AAVPHP.B_Igk-TATk-CDKL5 vector had a different tropism in the various brain regions. By comparing the *CDKL5* mRNA levels in GT-treated *Cdkl5* +/− mice to those of wild-type mice, we found a significant recovery of CDKL5 expression in the cortex (Fig. 6A), thalamus (Fig. 6B), and brain stem (Fig. 6D and Supplementary Fig. 2A), and a positive trend in the hippocampus (Fig. 6E and F) and cerebellum (Supplementary Fig. 2B).

Unlike the wild-type condition, virus-infected cells exhibited heterogeneous *CDKL5* mRNA expression, with some cells showing very high levels of *CDKL5* mRNA (Fig. 6C and Supplementary Figs. 1 and 2). To detect whether CDKL5 overexpression is harmful to brain cells, we evaluated the nuclear morphology of cells expressing high levels of *CDKL5* mRNA. No pyknotic nuclei were observed (Supplementary Fig. 2C), indicating that high level expressing cells were not undergoing apoptosis.

In line with the absence or low CDKL5 expression in astrocytes [2,42], we did not detect CDKL5 expression, or found it at very low levels, in GFAP-positive cells in brain slices subjected to in situ hybridization (Supplementary Fig. 3). Although AAV-PHP.B can infect astrocytes, albeit less efficiently than neurons [43,44], we identified a small number of astrocytes expressing CDKL5 in the brains of GT-treated *Cdkl5* +/− mice (Supplementary Fig. 3) that could be interpreted as expressing virally delivered CDKL5. This observation supports the idea that, even if CDKL5 is expressed in glial cells, typically devoid of it, no significant harmful effects are observed. Thus, while CDKL5 expression may occur in astrocytes, viral delivery of CDKL5 appears safe, reinforcing the safety of gene therapy in these cells (Fig. 5F and G).

Recovery of *CDKL5* mRNA expression was confirmed by the CDKL5 protein levels in the brain of GT-treated *Cdkl5* +/− mice, evaluated through immunofluorescence (Fig. 6G and Supplementary Fig. 4B) and Western blot analysis (Supplementary Fig. 4A). Reflecting the increase in CDKL5 protein expression, the phosphorylation levels of the direct CDKL5 target, EB2, was recovered (Fig. 6H).

### Effect of gene therapy in peripheral tissues of heterozygous *Cdkl5* +/− female mice

**Fig. 7:**
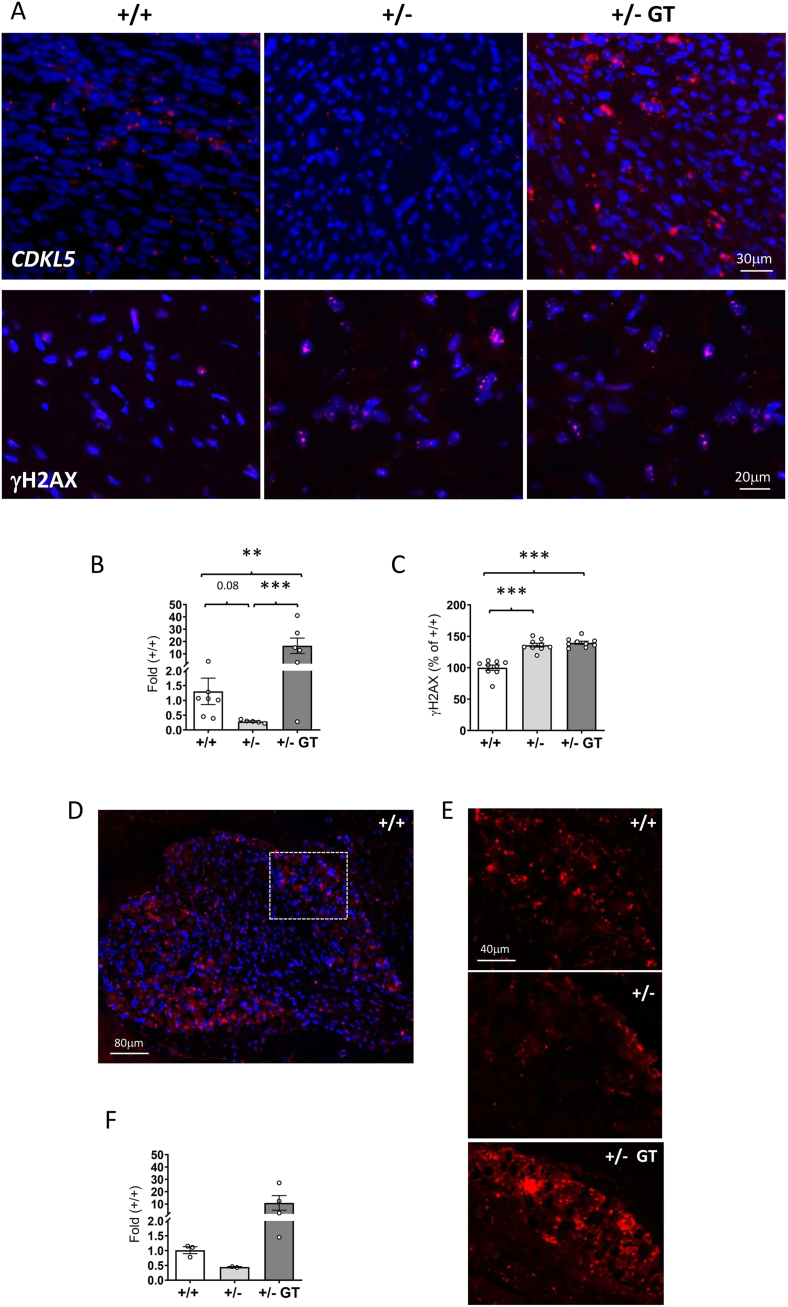
**TATk-CDKL5 expression in the heart and DRG of middle-aged *Cdkl5*** ​+/− ​**mice. A:** Upper panel: representative images of ventricular sections with fluorescence in situ hybridization (ISH) for *CDKL5* mRNA (red) of a wild-type (+/+) and a *Cdkl5*(+/−) mouse, and of a *Cdkl5* ​mouse treated with the Igk-TATk-CDKL5 vector (+/− ​GT). Scale bar ​= ​30 ​μm. Lower panel: Representative fluorescent images of ventricular tissue sections immunostained for γH2AX (red) and counterstained with DAPI (blue) from a wild-type (+/+) and a *Cdkl5* ​mouse (+/−), and from a *Cdkl5* ​mouse treated with the Igk-TATk-CDKL5 vector (+/− ​GT). Scale bar ​= ​20 ​μm. **B:** Real-time qPCR analysis of the levels of *CDKL5* mRNA expression in the heart of wild-type mice (+/+; *n* ​= ​7) and *Cdkl5* ​mice (+/−; *n* ​= ​5), and of *Cdkl5* ​mice treated with the Igk-TATk-CDKL5 vector (+/− ​GT; *n* ​= ​6). Data are given as fold change in comparison with *CDKL5* mRNA expression levels in the heart of wild-type mice. **C:** Quantification of γH2AX nuclear signal intensity per area in the cardiac tissue of wild-type (+/+; *n* ​= ​9) and *Cdkl5* (+/−; *n* ​= ​9) mice, and of *Cdkl5* ​mice treated with the Igk-TATk-CDKL5 vector (+/− ​GT; *n* ​= ​9). Data are expressed as a percentage of the wild-type condition. **D:** Representative image of a dorsal root ganglia (DRG) section with fluorescence ISH for *CDKL5* mRNA (red) and counterstained with DAPI (blue) of a wild-type (+/+) mouse. The dashed white box indicates the DRG region, which is shown at higher magnification in the various panels in E, corresponding to the different experimental conditions. **E**: Representative images of DRG sections with ISH for *CDKL5* mRNA (red) of a wild-type (+/+) and a *Cdkl5* ​(+/−) ​mouse, and of a *Cdkl5* ​mouse treated with the Igk-TATk-CDKL5 vector (+/− ​GT). **F:** Real-time qPCR analysis of the levels of *CDKL5* mRNA expression in the DRG of wild-type (+/+; *n* ​= ​3) and *Cdkl5* ​(+/−; *n* ​= ​2) mice, and of *Cdkl5* ​mice treated with the Igk-TATk-CDKL5 vector (+/− ​GT; *n* ​= ​4). Data are given as fold change in comparison with *CDKL5* mRNA expression levels in the DRG of wild-type mice. Values in B,C,F represent means ​± ​SEM. ∗*p* ​< ​0.05, ∗∗∗*p* ​< ​0.001. Fisher's LSD test after one-way ANOVA.

Although *Cdkl5* +/− mice received GT administered via intracarotid injection, virus particles can spread from the CNS to peripheral tissues, particularly to the heart (Fig. 7A), which is the first tissue that receives the blood flowing from the brain. Given the limited sensitivity of ISH in tissues with low *CDKL5* expression and the lack of reliable antibodies for CDKL5 immunohistochemistry, we focused our analyses of these regions primarily on qRT-PCR. Albeit at lower levels than in the brain [37], *CDKL5,* quantified by qRT-PCR*,* was expressed in the mouse heart, and reduced to approximately 30 % in heterozygous *Cdkl5* +/− female mice in comparison with wild-type mice (Fig. 7B). GT-treated *Cdkl5* +/− mice showed an increased *CDKL5* expression in the heart, which reached levels that were even 15–20 times higher than that of the wild-type mice (Fig. 7B), with a distribution throughout the thickness of the cardiac wall (Supplementary Fig. 5). To assess whether CDKL5 overexpression has harmful effects at the cardiac level, we quantified a marker of oxidative stress, γH2AX [45]. The γH2AX signal detection is regularly used to visualize and quantify the extent of double-strand breaks (DSBs) to monitor DNA damage, aging, and cellular health. We found higher γH2AX levels in the *Cdkl5* +/− heart in comparison with wild-types (Fig. 7A and C), which is indicative of increased ROS production in the absence of CDKL5 [37]. Importantly, GT treatment did not affect γH2AX levels in the heart of *Cdkl5* +/− mice (Fig. 7A and C).

To determine if the re-expression of CDKL5 within the dorsal root ganglia (DRG), where primary nociceptors are localized, contributes to the observed restoration of pain perception in *Cdkl5* +/− mice, *CDKL5* levels were quantified by qRT-PCR. As previously demonstrated [38] *CDKL5* is expressed in the sensory neurons of the DRG (Fig. 7D–F), suggesting its role in influencing the function and sensitivity of nociceptive neurons. Although the comparison did not reach statistical significance due to low sample size and high intra-group variability in the treated group, a clear biological trend was observed. As expected, in heterozygous *Cdkl5* +/− female mice *CDKL5* expression was reduced by 50 % compared to wild-type mice (Fig. 7E and F). Following GT treatment, *CDKL5* expression in the DRG was increased, with several treated animals reaching or exceeding wild-type *CDKL5* mRNA levels, indicating that the virus particles successfully reached the DRGs.

Importantly, no inflammatory reactions, as assessed by the levels of plasma pro-inflammatory cytokines (TNFα, IL-6, IL-1β) and the anti-inflammatory cytokine (IL-10), were observed in GT-treated *Cdkl5* +/− mice (Table 1), suggesting the absence of any generalized negative effects due to CDKL5 overexpression.

**Table 1:** **Effect of Igk-TATk-CDKL5 gene therapy on plasma cytokine levels in heterozygous *Cdkl5* female mice**. Circulating levels of cytokines (TNF-α, IL-6, IL-1β, IL-10) were evaluated in the plasma of wild-type (+/+; n = 14) and heterozygous *Cdkl5* (+/−; n = 12) mice, and heterozygous *Cdkl5* mice treated with the Igk-TATk-CDKL5 vector (+/− GT; n = 9). Values are presented as mean ± SEM. n.s.: no significant differences between groups. Fisher's LSD test after one-way ANOVA.

Cytokines	Plasma level (pg/ml)			
	+/+	+/−	+/− GT	Significant
**TNFα**	7.05 ​± ​1.10	4.09 ​± ​0.58	4.23 ​± ​0.66	n.s.
**IL6**	44.84 ​± ​5.69	34.62 ​± ​5.28	35.96 ​± ​4.88	n.s.
**IL1β**	17.22 ​± ​2.53	13.20 ​± ​2.83	20.39 ​± ​2.90	n.s.
**IL10**	76.54 ​± ​12.54	66.03 ​± ​9.22	76.47 ​± ​14.35	n.s.

## Discussion

Gene and protein replacement therapies have emerged as promising strategies for addressing CDKL5 Deficiency Disorder (CDD) [20,21,46]. Recently, we developed a hybrid approach that integrates key features of gene therapy and protein replacement therapy to restore CDKL5 function in the brain [21]. Specifically, we engineered a virally delivered transgene encoding a secreted, cell-penetrant form of CDKL5 protein, which has the potential to not only correct transduced cells but also neighboring cells through a cross-correction mechanism. In our prior work [21], we presented evidence supporting the potential efficacy of this approach, showing that cross-correction could offer therapeutic advantages over conventional gene delivery methods. However, an emerging concern for both protein and gene replacement lies in the sensitivity of cells to CDKL5 dosage, particularly when reintroducing the protein into wild-type cells, a scenario that is highly relevant to female CDD patients with mosaic loss of CDKL5, or in tissues with low or absent CDKL5 expression. Supraphysiological CDKL5 levels could compromise therapeutic benefit or even exacerbate disease manifestations. Here, we provide compelling evidence supporting the efficacy and safety of a cross-correction-based gene therapy approach in *Cdkl5* heterozygous female mice. Our findings demonstrate that re-expression of CDKL5 can effectively ameliorate disease-related phenotypes in a mosaic neuronal environment, without inducing adverse effects associated with protein overdosage. These results strengthen the translational potential of cross-correction-based therapies for treating CDD, especially in female patients with mosaicism. While these findings suggest the promise of this approach, further direct evidence is needed to conclusively demonstrate the mechanism of cross-correction in this genetically complex setting.

To compare the effectiveness of GT on the CDD-related phenotype in hemizygous *Cdkl5* KO males and heterozygous *Cdkl5* +/− females, we employed the same viral dose (10^12^ vg/mouse), and route of administration (systemic, via intracarotid injection) established in our previous work [21]. Importantly, *Cdkl5* +/− mice aged 10–12 months were selected in an attempt to evaluate the GT effect on the epileptic phenotype that characterizes aged female *Cdkl5* +/− mice [16,17]. To fully exploit the potential of a cross-correction strategy, where both neuronal and non-neuronal cells can contribute to therapeutic protein production, we drove expression of Igk-TATk-CDKL5 under the control of the ubiquitous CBh promoter [47].

Table 2 provides a comparative analysis between GT-induced behavioral improvements between male *Cdkl5* KO mice [21] and heterozygous female *Cdkl5* +/− mice from the present study. Notably, female *Cdkl5* +/− mice exhibited behavioral defects of comparable severity to those observed in male *Cdkl5* -/Y mice (Table 2), consistent with the similar clinical manifestations reported in male and female CDD patients [4]. However, we cannot exclude the possibility that this comparative analysis between male (5-6-month) and female (10-12-month) *Cdkl5* KO mice may be influenced by the differing mouse age. Indeed, a worsening of the behavioral phenotype with age was demonstrated in *Cdkl5* KO male mice [48], potentially influencing the degree of phenotypic severity observed.

**Table 2:** **Efficacy of Igk-TATk-CDKL5 gene therapy on behavior in male and female *Cdkl5* KO mice**. Comparison of the results obtained in different behavioral tests between heterozygous *Cdkl5* female mice (+/−) and hemizygous *Cdkl5* male mice (−/Y) following treatment with Igk-TATk-CDKL5 gene therapy (GT). Data are expressed as a percentage of the wild-type condition. A check mark indicates a significant difference compared to the KO condition of the same gender. OF: Open Field; n.d. = not determined.

Behavioral tests	Genotype					
	Females			Males		
	+/+	+/−	+/− GT	+/Y	−/Y	−/Y GT
**Marble burying**	100.00 ​± ​3.63	45.31 ​± ​8.83	54.14 ​± ​11.16	100.00 ​± ​2.44	55.40 ​± ​10.00	74.96 ​± ​6.70
**Nesting test**	100.00 ​± ​5.95	58.13 ​± ​4.76	89.23 ​± ​10.56**✓**	100.00 ​± ​7.28	57.95 ​± ​3.91	78.55 ​± ​6.62**✓**
**Clasping of hind limbs** *Time in sec*	100.00 ​± ​48.06	445.08 ​± ​97.10	676.36 ​± ​56.91	100.00 ​± ​36.75	530.25 ​± ​67.33	497.95 ​± ​43.12
**OF: Distance moved** *0-*5 ​min	100.00 ​± ​8.24	141.00 ​± ​11.57	111.32 ​± ​11.93**✓**	100.00 ​± ​4.40	101.24 ​± ​14.30	90.66 ​± ​5.55
**OF: Distance moved** *5-*10 ​min	100.00 ​± ​7.09	135.93 ​± ​8.98	111.30 ​± ​9.83	100.00 ​± ​5.46	136.20 ​± ​17.30	111.46 ​± ​4.45**✓**
**OF: Distance moved** *10-*15 ​min	100.00 ​± ​6.18	122.55 ​± ​8.92	115.59 ​± ​9.90	100.00 ​± ​5.34	147.20 ​± ​17.31	120.84 ​± ​4.05
**OF: Velocity** *0-*5 ​min	100.00 ​± ​8.24	141.05 ​± ​11.58	111.28 ​± ​11.93**✓**	100.00 ​± ​4.39	101.14 ​± ​14.22	90.59 ​± ​5.55
**OF: Velocity** *5-*10 ​min	100.00 ​± ​7.09	136.05 ​± ​9.02	111.40 ​± ​9.84	100.00 ​± ​5.50	136.02 ​± ​17.27	111.25 ​± ​4.45**✓**
**OF: Velocity** *5-*10 ​min	100.00 ​± ​6.17	122.58 ​± ​8.95	115.60 ​± ​9.89	100.00 ​± ​5.36	146.95 ​± ​17.32	120.72 ​± ​4.06
**OF: Stereotypic jumps** *score*	100.00 ​± ​26.83	307.36 ​± ​48.46	129.42 ​± ​59.91**✓**	100.00 ​± ​76.38	3620 ​± ​1779	1089 ​± ​716
**Rotarod: Passive rotations** *Frequency*	100.00 ​± ​32.97	872.24 ​± ​180.68	280.51 ​± ​80.14**✓**	100.00 ​± ​44.5	791.2 ​± ​200	847.4 ​± ​227
**Barnes maze learning** *Day 1*	100.00 ​± ​11.71	171.85 ​± ​19.38	131.24 ​± ​18.26**✓**	100.00 ​± ​16.40	125.96 ​± ​17.41	116.55 ​± ​14.76
**Barnes maze learning** *Day 2*	100.00 ​± ​14.32	207.80 ​± ​30.85	180.10 ​± ​22.42	100.00 ​± ​18.28	355.71 ​± ​46.43	236.47 ​± ​41.02**✓**
**Barnes maze learning** *Day 3*	100.00 ​± ​16.82	233.98 ​± ​30.11	182.58 ​± ​25.35	100.00 ​± ​18.32	544.19 ​± ​63.44	366.84 ​± ​72.03
**Barnes maze memory** *Day 4*	100.00 ​± ​14.06	380.38 ​± ​60.37	179.19 ​± ​32.19**✓**	100.00 ​± ​32.07	278.74 ​± ​43.03	288.84 ​± ​45.87
**Mechanical allodynia** *Latency*	100.00 ​± ​9.59	52.21 ​± ​5.70	91.09 ​± ​17.18**✓**	n.d.	n.d.	n.d.
**Mechanical allodynia** *Force*	100.00 ​± ​7.27	64.98 ​± ​3.64	86.48 ​± ​9.30**✓**	n.d.	n.d.	n.d.

Regarding the effect of GT, here we show that several CDD-related phenotypes, including nesting behavior, stereotyped behavior, motor coordination, and memory performance, were significantly improved in treated *Cdkl5* +/− female mice. Hyperactivity also exhibited a partial improvement. In contrast, we found no improvement in marble burying or hind-limb clasping, suggesting that distinct behavioral domains may be differentially sensitive to the CDKL5 re-expression. Notably, at the same viral dose, heterozygous *Cdkl5* +/− mice showed a greater behavioral improvement than *Cdkl5* −/Y mice, particularly in innate behaviors, stereotypic movements, motor function, and hippocampal-dependent memory performance (Table 2). The higher efficacy of GT observed in the heterozygous condition likely reflects the lower number of neurons (∼50%) requiring CDKL5 restoration compared to the complete deficiency present in hemizygous males (100%).

In our study, we observed significant behavioral improvements in the gene therapy-treated group, including enhanced motor and cognitive functions. However, it is important to note that, since aged heterozygous *Cdkl5* females are known to experience spontaneous seizures, albeit infrequently, prior seizure activity may have influenced the behavioral assessments. This potential confounder should be taken into account when interpreting our results. Further studies specifically investigating the impact of seizure activity on behavior are needed to better understand the true therapeutic effect.

Anamnestic evidence indicates that CDD patients have altered nociception [38], with either reduced or increased perception. Here we report that *Cdkl5* +/− female mice show increased sensitivity to pain generated from mechanical stimuli. Gender differences in pain perception in both humans and in animals have been demonstrated [49,50]; this may explain the recently described difference in pain response of male *Cdkl5* KO mice, with reduced nociception [38]. The finding that GT treatment restored pain perception in *Cdkl5* +/− female mice suggests that heightened pain sensitivity in the heterozygous CDKL5 condition may be linked to dysfunctional cortical pain processing or impaired primary peripheral nociception. Indeed, increased CDKL5 expression was also observed in the dorsal root ganglia of GT-treated mice, further supporting the idea that enhancing CDKL5 expression can positively impact pain processing pathways. This further confirm that CDKL5 expression plays a critical role in modulating pain perception, and restoring its function in both central and peripheral systems could potentially correct pain sensitivity in patients with CDD.

Consistent with previous reports [16,17], we observed spasms in *Cdkl5* +/− mice associated to interictal electrographic activity. These seizures were mainly associated to stiffening of the tail, resembling Straub tail (a motor behavior associated to seizures in other mouse models of X-linked mental retardation such as Fragile X syndrome [39]), or facial automatisms according to a validated scale [32]. Although we also observed tonic-clonic seizures in *Cdkl5* +/− female mice, the very low frequency of these events unfortunately precluded their detection during the 48-h baseline EEG recordings. It is worth to note, however, that the epilepsy prevalence was not different between wild-type and *Cdkl5* +/− mice; this may be due to the old age of mice suggesting that at least some of these seizures were age-dependent idiopathic seizures [[51], [52], [53], [54], [55], [56]].

Although a recent observation suggested that late restoration of Cdkl5, using a genetic strategy, did not rescue CDD-related electrographic changes [57], we observed that the elevated delta power in *Cdkl5* +/− mice is reverted to the wild-type condition by GT treatment. Since elevated delta spectral power correlated with seizures in *Cdkl5* +/− mice [17], and is present in CDD patients [58], its recovery suggests a positive impact of GT on CDD-dependent EEG abnormalities. However, we currently have no indications as to whether the phenotype of severe debilitating (tonic-clonic) seizures in *Cdkl5* KO mice is reversible by GT treatment. In future studies, a more prolonged video-EEG recording will allow for a detailed characterization of the impact of GT on the seizure phenotype in *Cdkl5* +/− mice. As expected, when mice were monitored under stress-inducing conditions, such as the sleep post-deprivation, seizure phenotype worsened; in particular, the seizure duration increased. Interestingly, restoration of CDKL5 by GT in *Cdkl5* +/− mice was shown to shorten seizure duration to levels comparable to those observed in baseline conditions.

As previously reported in male *Cdkl5*KO mice [21], several neuroanatomical alterations, including impaired dendritic and synaptic development, hippocampal neuronal survival, and microglia over-activation were strongly ameliorated, or even restored, in GT-treated female *Cdkl5* +/− mice (Table 3). The improvement in neuroanatomical defects was more pronounced than the improvement observed in behavioral performances (Table 2), suggesting that other neuronal alterations, not yet known or not investigated here, may contribute to certain behavioral deficits and are only partially corrected by GT treatment. However, we cannot also exclude the possibility that some CDD-related behavioral impairments are irreversible, or only partially reversible, in adult *Cdkl5* KO mice.

**Table 3:** **Efficacy of Igk-TATk-CDKL5 gene therapy on neuroanatomy in male and female *Cdkl5* KO mice**. Comparison of the result obtained in different neuroanatomical analyses between heterozygous *Cdkl5* female mice (+/−) and hemizygous *Cdkl5* male mice (−/Y) following treatment with Igk-TATk-CDKL5 gene therapy (GT). Data are expressed as a percentage of the wild-type condition. A check mark indicates a significant difference compared to the KO condition of the same gender. ^C^: cortical; ^H^: hippocampal; n.d. ​= ​not determined.

Neuroanatomy	Genotype					
	Females			Males		
	+/+	+/−	+/− GT	+/Y	−/Y	−/Y GT
**Basal dendrites** *Total length*	100.00 ​± ​3.57**^C^**	84.46 ​± ​2.29**^C^**	92.17 ​± ​2.81**^C^✓**	100.00 ​± ​1.13**^H^**	85.95 ​± ​2.22**^H^**	100.75 ​± ​2.52**^H^✓**
**Basal dendrites** *N° of branches*	100.00 ​± ​2.85**^C^**	89.66 ​± ​2.96**^C^**	100.03 ​± ​2.89**^C^✓**	100.00 ​± ​0.44**^H^**	81.03 ​± ​6.04**^H^**	96.62 ​± ​4.19**^H^✓**
**Basal dendrites** *Mean length*	100.00 ​± ​6.60**^C^**	85.59 ​± ​2.20**^C^**	93.78 ​± ​2.06 ​**^C^**	100.00 ​± ​3.39**^H^**	87.86 ​± ​4.86**^H^**	101.87 ​± ​3.25**^H^✓**
**Dendritic spines** *Density*	100.00 ​± ​1.32**^C^**	93.71 ​± ​1.05**^C^**	98.87 ​± ​0.50**^C^✓**	100.00 ​± ​2.19**^H^**	79.43 ​± ​2.59**^H^**	95.56 ​± ​1.80**^H^✓**
**Dendritic spines** *% of mature*	100.00 ​± ​1.59**^C^**	72.69 ​± ​1.92**^C^**	93.99 ​± ​4.66**^C^✓**	100.00 ​± ​3.84**^H^**	55.22 ​± ​5.65**^H^**	95.68 ​± ​3.50**^H^✓**
**PSD-95 puncta** *Cortical density*	100.00 ​± ​1.84	83.47 ​± ​1.08	98.95 ​± ​2.22 **✓**	n.d.	n.d.	n.d.
**PSD-95 puncta** *Hippocampal density*	100.00 ​± ​0.94	82.46 ​± ​1.51	91.59 ​± ​1.58 **✓**	100.01 ​± ​1.55	71.76 ​± ​1.67	98.36 ​± ​0.59**✓**
**AIF-1 body size** *Cortex*	100.09 ​± ​1.09	104.66 ​± ​0.89	98.16 ​± ​1.67**✓**	100.09 ​± ​0.90	113.11 ​± ​1.11	100.69 ​± ​1.21**✓**
**AIF-1 body size** *Hippocampus*	100.00 ​± ​0.36	105.18 ​± ​1.50	101.02 ​± ​0.50**✓**	n.d.	n.d.	n.d.
**AIF-1 cell density** *Cortex*	100.00 ​± ​3.14	99.61 ​± ​1.88	95.71 ​± ​5.15	100.00 ​± ​0.43	95.98 ​± ​7.31	99.82 ​± ​3.11
**AIF-1 cell density***Hippocampus*	100.00 ​± ​2.07	103.63 ​± ​3.18	99.28 ​± ​3.80	n.d.	n.d.	n.d.
**DAPI ​+ ​cells** *Hippocampal CA1*	100.00 ​± ​1.54	91.73 ​± ​0.46	98.72 ​± ​0.67**✓**	100.00 ​± ​1.50	89.80 ​± ​0.63	98.95 ​± ​0.60**✓**
**NeuN ​+ ​cells** *Hippocampal CA1*	n.d.	n.d.	n.d.	100.00 ​± ​0.76	89.71 ​± ​1.62	102.03 ​± ​1.55**✓**

Furthermore, the quantification of the number of *CDKL5* mRNA positive cells indicated a partial recovery of CDKL5 re-expression, which could underlie the limited improvement in some behaviors. However, this analysis reflects only the number of infected cells and does not account for cells receiving CDKL5 protein through cross-correction mechanism. Regarding the safety of this therapeutic approach, as previously reported [21], GT-treated *Cdkl5* +/− mice showed no signs of neuroinflammatory responses against the TATk-CDKL5 protein, as assessed by microglial cell count, nor alterations in body weight, an indicator of overall well-being.

To further evaluate the safety of this therapeutic approach, we examined the effects of gene therapy on wake-sleep behavior, given the close relationship between sleep quality and overall health and disease. Our findings demonstrate that CDKL5 deficiency in heterozygous female mice leads to disrupted sleep architecture, particularly during the light phase. The significant reduction in wakefulness observed in female *Cdkl5* +/− mice during the light phase, coupled with a corresponding increase in NREM sleep, may reflect impaired regulation of arousal and sleep-wake transitions. Since the light phase corresponds to the resting period in nocturnal animals, this alteration suggests a deficit in the ability to appropriately modulate sleep pressure and maintain stable wakefulness during their inactive phase. Notably, despite the absence of observable changes in overall sleep fragmentation, heterozygous mice exhibited a decreased number of NREM episodes over a 24-h period and a shorter average wake bout duration, suggesting reduced state transitions and more consolidated but dysregulated sleep. This phenotype may represent a preclinical correlate of the sleep disturbances frequently reported in patients with CDD [5,59,60], including excessive sleepiness, disrupted sleep-wake rhythms, and difficulties in sustaining wakefulness.

Consistent with the involvement of CDKL5 in sleep regulation, a recent study in hemizygous male *Cdkl5* KO mice [61] reported a significant reduction in NREM sleep and increased wakefulness. Additionally, male KO mice exhibited increased sleep fragmentation, with a higher number of short-duration wake and NREM episodes. Interestingly, our findings in heterozygous female mice contrast with those observed in male *Cdkl5* KO mice. These differences may reflect sex-specific mechanisms of sleep control or compensatory adaptations to mosaic CDKL5 expression in females. Nevertheless, alterations in sleep architecture in *Cdkl5* +/− mice may provide valuable translational insights into the pathophysiology of sleep impairments associated with heterozygous CDKL5 mutations.

Interestingly, while gene therapy with Igk-TATk-CDKL5 did not restore normal sleep/wake proportions during the light phase, it successfully rescued the number of NREM episodes over the 24-h cycle, indicating an improvement in sleep-state switching. However, it did not fully restore arousal stability or the ability to sustain prolonged wakefulness. This finding may be due to the limited and region-specific transduction pattern of the AAV vector used for CDKL5 delivery, but we cannot exclude the possibility that, when therapy is applied postnatally or after certain circuits mature, it might not restore normal sleep pressure or circadian signaling. It is important to note, however, that the therapy did not induce significant changes in the percentage of time spent in each behavioral state. These findings align with previous results [21], which reported no differences in sleep architecture between GT-treated male *Cdkl5* KO mice and age-matched untreated controls. Therefore, the absence of significant differences in the sleep fragmentation index across experimental groups suggests that gene therapy does not exacerbate sleep disruption.

Importantly, cells with high *CDKL5* mRNA did not show morphological features of apoptosis, indicating that *CDKL5* overexpression is not detrimental to brain cells. Similarly, heart cells overexpressing *CDKL5* showed no increase in DNA damage, as measured by γH2AX levels. However, it is noteworthy that GT treatment in *Cdkl5* +/− heart did not restore γH2AX levels to those observed in the wild-type condition. This finding suggests that the increased oxidative stress and mitochondrial dysfunction reported in *Cdkl5* +/− hearts [37] are unlikely to result from CDKL5 deficiency in cardiomyocytes, further supported by the very low levels of CDKL5 expression in wild-type hearts [37].

Since inflammation represents the immune system's response to harmful stimuli, such as damaged cells or toxic compounds, our finding that levels of key pro- and anti-inflammatory cytokines were unaltered in GT-treated *Cdkl5* +/− mice strongly suggests an absence of peripheral toxicity associated with Igk-TATk-CDKL5. However, a limitation of this study is the lack of a comprehensive evaluation of the efficacy and safety of gene therapy (GT) treatment across peripheral tissues, including the kidney. A recent study has highlighted the activation of CDKL5 in acute kidney injury, where it contributes to signaling pathways leading to renal tubular epithelial cell dysfunction [62]. While it remains unclear whether CDKL5 overexpression could be harmful to healthy kidneys, future studies will be necessary to address this important question. Although our results suggest that CDKL5 overexpression does not cause significant toxicity within the scope of our current analyses, a more comprehensive safety evaluation would be required to make definitive conclusions regarding the safety of this approach. Such assessments will include the investigation of potential off-target effects, immune responses, and additional molecular markers of toxicity, all of which will be incorporated into future studies.

While these results demonstrate that gene therapy leads to significant improvements in several disease-related phenotypes in heterozygous female *Cdkl5* +/− mice, we acknowledge that full rescue was not achieved across all assays. These partial improvements are in line with previous studies [57], which suggest that gene restoration at later ages may have limitations. It is important to note that the gene therapy was administered at a single time point (10 months of age). This timing could be a critical factor influencing the extent of the therapeutic effect, as earlier intervention has been shown to yield stronger therapeutic outcomes compared to treatment at older ages [57]. Our findings could support that timing and age are key variables influencing the success of gene therapy in CDD, particularly in the context of older females with mosaic CDKL5 expression. Further studies across different age groups are necessary to better understand the full scope of therapeutic benefits and limitations associated with gene therapy in CDD.

## Conclusions

In conclusion, this study provides the first evidence that CDKL5 expression in a context of mosaic expression is both safe and effective in ameliorating neuroanatomical and behavioral defects characterizing the CDD mouse model. While studies emphasized the need for widespread vector distribution, requiring high viral doses, to achieve therapeutic efficacy [20], our findings demonstrate that a cross-correction based gene delivery approach can attain significant therapeutic benefits without the necessity for high vector doses. This strategy reduces the risk of dose-related toxicities, a very important consideration for future clinical translation. Therefore, we propose that a cross-correction gene therapy approach could be a promising and potentially safer option for treating CDD. However, whether the efficacy observed in the mouse model will translate successfully to human patients remains an open and critical question. Furthermore, while the Cdkl5 +/− mouse model offers a valuable platform for assessing overexpression toxicity in a context that more closely mirrors the condition in female CDD patients, it is important to note that a thorough safety evaluation should also involve wild-type mice treated with gene therapy. Future studies should incorporate wild-type animals to provide a comprehensive safety assessment in a non-diseased context, thereby establishing a clear and definitive safety profile necessary for regulatory processes, including FDA approval submissions.

## Data availability

The datasets analyzed during the current study are available from the corresponding author upon reasonable request.

## Author contributions

Conceptualization, E.C., S.T. and G.M.; Data curation, G.M., M.T., and E.C.; Formal analysis, G.M., M. T., M.L., A.M.B., B.S.G., G.V., N.M., G.M., V.L.M., C.B., G.C., F.T., A.R., P.S., and S.T.; Funding acquisition, E.C.; Investigation, G.M., and M.T.; Methodology, G.M., S.T., and E.C.; Supervision, E.C., G.Z., G.C. and G.M; Writing—original draft preparation, E.C. and G.M.; Writing—review and editing, E.C., G.M., and S.T. All authors have read and agreed to the published version of the manuscript.

## Declaration of Generative AI and AI-assisted technologies in the writing process

The authors used ChatGPT (OpenAI) solely to improve the English language and grammar of the manuscript. All scientific content, data interpretation, and conclusions were entirely conceived, written, and critically reviewed by the authors. The AI tool was not used for data analysis, result interpretation, or the generation of original scientific content. The authors take full responsibility for the content of this article.

## Declaration of competing interest

The authors declare the following financial interests/personal relationships which may be considered as potential competing interests: Elisabetta Ciani reports financial support was provided by Ministry of University and Research (MUR), National Recovery and Resilience Plan (NRRP). Elisabetta Ciani reports financial support was provided by CDKL5 - Associazione di volontariato Onlus. Elisabetta Ciani has patent #New Gene Therapy Constructs Patent Numbers: IT102019000008877; EP20730331.4; US20220175966; CN113950533 licensed to University of Bologna. If there are other authors, they declare that they have no known competing financial interests or personal relationships that could have appeared to influence the work reported in this paper.

## References

[1] Kalscheuer V.M., Tao J., Donnelly A., Hollway G., Schwinger E., Kubart S. (2003). Disruption of the serine/threonine kinase 9 gene causes severe X-linked infantile spasms and mental retardation. Am J Hum Genet.

[2] Rusconi L., Salvatoni L., Giudici L., Bertani I., Kilstrup-Nielsen C., Broccoli V. (2008). CDKL5 expression is modulated during neuronal development and its subcellular distribution is tightly regulated by the C-terminal tail. J Biol Chem.

[3] Bertani I., Rusconi L., Bolognese F., Forlani G., Conca B., De Monte L. (2006). Functional consequences of mutations in CDKL5, an X-linked gene involved in infantile spasms and mental retardation. J Biol Chem.

[4] Olson H.E., Demarest S.T., Pestana-Knight E.M., Swanson L.C., Iqbal S., Lal D. (2019). Cyclin-dependent kinase-like 5 deficiency disorder: clinical review. Pediatr Neurol.

[5] Fehr S., Wilson M., Downs J., Williams S., Murgia A., Sartori S. (2013). The CDKL5 disorder is an independent clinical entity associated with early-onset encephalopathy. Eur J Hum Genet.

[6] Leonard H., Downs J., Benke T.A., Swanson L., Olson H., Demarest S. (2022). CDKL5 deficiency disorder: clinical features, diagnosis, and management. Lancet Neurol.

[7] Demarest S.T., Olson H.E., Moss A., Pestana-Knight E., Zhang X., Parikh S. (2019). CDKL5 deficiency disorder: relationship between genotype, epilepsy, cortical visual impairment, and development. Epilepsia.

[8] Liang J.S., Huang H., Wang J.S., Lu J.F. (2019). Phenotypic manifestations between male and female children with CDKL5 mutations. Brain Dev.

[9] Wang I.T., Allen M., Goffin D., Zhu X., Fairless A.H., Brodkin E.S. (2012). Loss of CDKL5 disrupts kinome profile and event-related potentials leading to autistic-like phenotypes in mice. Proc Natl Acad Sci USA.

[10] Amendola E., Zhan Y., Mattucci C., Castroflorio E., Calcagno E., Fuchs C. (2014). Mapping pathological phenotypes in a mouse model of CDKL5 disorder. PLoS One.

[11] Okuda K., Kobayashi S., Fukaya M., Watanabe A., Murakami T., Hagiwara M. (2017). CDKL5 controls postsynaptic localization of GluN2B-containing NMDA receptors in the hippocampus and regulates seizure susceptibility. Neurobiol Dis.

[12] Tang S., Terzic B., Wang I.J., Sarmiento N., Sizov K., Cui Y. (2019). Altered NMDAR signaling underlies autistic-like features in mouse models of CDKL5 deficiency disorder. Nat Commun.

[13] Wang H.T., Zhu Z.A., Li Y.Y., Lou S.S., Yang G., Feng X. (2021). CDKL5 deficiency in forebrain glutamatergic neurons results in recurrent spontaneous seizures. Epilepsia.

[14] Fuchs C., Rimondini R., Viggiano R., Trazzi S., De Franceschi M., Bartesaghi R. (2015). Inhibition of GSK3beta rescues hippocampal development and learning in a mouse model of CDKL5 disorder. Neurobiol Dis.

[15] Fuchs C., Gennaccaro L., Trazzi S., Bastianini S., Bettini S., Lo Martire V. (2018). Heterozygous CDKL5 knockout female mice are a valuable animal model for CDKL5 disorder. Neural Plast.

[16] Terzic B., Cui Y., Edmondson A.C., Tang S., Sarmiento N., Zaitseva D. (2021). X-linked cellular mosaicism underlies age-dependent occurrence of seizure-like events in mouse models of CDKL5 deficiency disorder. Neurobiol Dis.

[17] Mulcahey P.J., Tang S., Takano H., White A., Davila Portillo D.R., Kane O.M. (2020). Aged heterozygous Cdkl5 mutant mice exhibit spontaneous epileptic spasms. Exp Neurol.

[18] Gao Y., Irvine E.E., Eleftheriadou I., Naranjo C.J., Hearn-Yeates F., Bosch L. (2020). Gene replacement ameliorates deficits in mouse and human models of cyclin-dependent kinase-like 5 disorder. Brain.

[19] Benke T.A., Kind P.C. (2020). Proof-of-concept for a gene replacement approach to CDKL5 deficiency disorder. Brain.

[20] Voronin G., Narasimhan J., Gittens J., Sheedy J., Lipari P., Peters M. (2024). Preclinical studies of gene replacement therapy for CDKL5 deficiency disorder. Mol Ther.

[21] Medici G., Tassinari M., Galvani G., Bastianini S., Gennaccaro L., Loi M. (2022). Expression of a secretable, cell-penetrating CDKL5 protein enhances the efficacy of gene therapy for CDKL5 deficiency disorder. Neurotherapeutics.

[22] Thorson L., Bryke C., Rice G., Artzer A., Schilz C., Israel J. (2010). Clinical and molecular characterization of overlapping interstitial Xp21-p22 duplications in two unrelated individuals. Am J Med Genet A.

[23] Szafranski P., Golla S., Jin W., Fang P., Hixson P., Matalon R. (2014). Neurodevelopmental and neurobehavioral characteristics in males and females with CDKL5 duplications. Eur J Hum Genet.

[24] Sismani C., Anastasiadou V., Kousoulidou L., Parkel S., Koumbaris G., Zilina O. (2011). 9 Mb familial duplication in chromosome band Xp22.2-22.13 associated with mental retardation, hypotonia and developmental delay, scoliosis, cardiovascular problems and mild dysmorphic facial features. Eur J Med Genet.

[25] Deacon R.M. (2006). Assessing nest building in mice. Nat Protoc.

[26] Yamamoto H., Shimoshige Y., Yamaji T., Murai N., Aoki T., Matsuoka N. (2009). Pharmacological characterization of standard analgesics on mechanical allodynia in streptozotocin-induced diabetic rats. Neuropharmacology.

[27] Bastianini S., Alvente S., Berteotti C., Lo Martire V., Matteoli G., Miglioranza E. (2025). Ageing-related modification of sleep and breathing in orexin-knockout narcoleptic mice. J Sleep Res.

[28] Malow B.A. (2004). Sleep deprivation and epilepsy. Epilepsy Curr.

[29] Bastianini S., Berteotti C., Gabrielli A., Lo Martire V., Silvani A., Zoccoli G. (2015). Recent developments in automatic scoring of rodent sleep. Arch Ital Biol.

[30] Bastianini S., Lo Martire V., Alvente S., Berteotti C., Matteoli G., Rullo L. (2021). Early-life nicotine or cotinine exposure produces long-lasting sleep alterations and downregulation of hippocampal corticosteroid receptors in adult mice. Sci Rep.

[31] Hirsch L.J., Fong M.W.K., Leitinger M., LaRoche S.M., Beniczky S., Abend N.S. (2021). American clinical neurophysiology society's standardized critical care EEG terminology: 2021 version. J Clin Neurophysiol.

[32] Veliskova J., Velisek L., Mares P., Rokyta R. (1990). Ketamine suppresses both bicuculline- and picrotoxin-induced generalized tonic-clonic seizures during ontogenesis. Pharmacol Biochem Behav.

[33] Fuchs C., Trazzi S., Torricella R., Viggiano R., De Franceschi M., Amendola E. (2014). Loss of CDKL5 impairs survival and dendritic growth of newborn neurons by altering AKT/GSK-3beta signaling. Neurobiol Dis.

[34] Galvani G., Mottolese N., Gennaccaro L., Loi M., Medici G., Tassinari M. (2021). Inhibition of microglia overactivation restores neuronal survival in a mouse model of CDKL5 deficiency disorder. J Neuroinflammation.

[35] Guidi S., Stagni F., Bianchi P., Ciani E., Ragazzi E., Trazzi S. (2013). Early pharmacotherapy with fluoxetine rescues dendritic pathology in the Ts65Dn mouse model of down syndrome. Brain Pathol.

[36] Tassinari M., Uguagliati B., Trazzi S., Cerchier C.B., Cavina O.V., Mottolese N. (2023). Early-onset brain alterations during postnatal development in a mouse model of CDKL5 deficiency disorder. Neurobiol Dis.

[37] Loi M., Bastianini S., Candini G., Rizzardi N., Medici G., Papa V. (2023). Cardiac functional and structural abnormalities in a mouse model of CDKL5 deficiency disorder. Int J Mol Sci.

[38] La Montanara P., Hervera A., Baltussen L.L., Hutson T.H., Palmisano I., De Virgiliis F. (2020). Cyclin-dependent-like kinase 5 is required for pain signaling in human sensory neurons and mouse models. Sci Transl Med.

[39] Curia G., Gualtieri F., Bartolomeo R., Vezzali R., Biagini G. (2013). Resilience to audiogenic seizures is associated with p-ERK1/2 dephosphorylation in the subiculum of Fmr1 knockout mice. Front Cell Neurosci.

[40] Wojcik S.M., Rhee J.S., Herzog E., Sigler A., Jahn R., Takamori S. (2004). An essential role for vesicular glutamate transporter 1 (VGLUT1) in postnatal development and control of quantal size. Proc Natl Acad Sci USA.

[41] Tassinari M., Mottolese N., Galvani G., Ferrara D., Gennaccaro L., Loi M. (2022). Luteolin treatment ameliorates brain development and behavioral performance in a mouse model of CDKL5 deficiency disorder. Int J Mol Sci.

[42] Silvestre M., Dempster K., Mihaylov S.R., Claxton S., Ultanir S.K. (2024). Cell type-specific expression, regulation and compensation of CDKL5 activity in mouse brain. Mol Psychiatr.

[43] Deverman B.E., Pravdo P.L., Simpson B.P., Kumar S.R., Chan K.Y., Banerjee A. (2016). Cre-dependent selection yields AAV variants for widespread gene transfer to the adult brain. Nat Biotechnol.

[44] Torregrosa T., Lehman S., Hana S., Marsh G., Xu S., Koszka K. (2021). Use of CRISPR/Cas9-mediated disruption of CNS cell type genes to profile transduction of AAV by neonatal intracerebroventricular delivery in mice. Gene Ther.

[45] Schutz C.S., Stope M.B., Bekeschus S. (2021). H2A.X phosphorylation in oxidative stress and risk assessment in plasma medicine. Oxid Med Cell Longev.

[46] Trazzi S., De Franceschi M., Fuchs C., Bastianini S., Viggiano R., Lupori L. (2018). CDKL5 protein substitution therapy rescues neurological phenotypes of a mouse model of CDKL5 disorder. Hum Mol Genet.

[47] Gray S.J., Foti S.B., Schwartz J.W., Bachaboina L., Taylor-Blake B., Coleman J. (2011). Optimizing promoters for recombinant adeno-associated virus-mediated gene expression in the peripheral and central nervous system using self-complementary vectors. Hum Gene Ther.

[48] Gennaccaro L., Fuchs C., Loi M., Pizzo R., Alvente S., Berteotti C. (2021). Age-related cognitive and motor decline in a mouse model of CDKL5 deficiency disorder is associated with increased neuronal senescence and death. Aging Dis.

[49] Vacca V., Marinelli S., Pieroni L., Urbani A., Luvisetto S., Pavone F. (2014). Higher pain perception and lack of recovery from neuropathic pain in females: a behavioural, immunohistochemical, and proteomic investigation on sex-related differences in mice. Pain.

[50] Pieretti S., Di Giannuario A., Di Giovannandrea R., Marzoli F., Piccaro G., Minosi P. (2016). Gender differences in pain and its relief. Ann Ist Super Sanita.

[51] D'Ambrosio R., Fender J.S., Fairbanks J.P., Simon E.A., Born D.E., Doyle D.L. (2005). Progression from frontal-parietal to mesial-temporal epilepsy after fluid percussion injury in the rat. Brain.

[52] Pena-Ceballos J., Moloney P.B., Choekyi T., Naggar H.E., Widdess-Walsh P., Delanty N. (2025). The clinical profile of adult-onset idiopathic generalised epilepsy. Seizure.

[53] Cutting S., Lauchheimer A., Barr W., Devinsky O. (2001). Adult-onset idiopathic generalized epilepsy: clinical and behavioral features. Epilepsia.

[54] Loiseau J., Crespel A., Picot M.C., Duche B., Ayrivie N., Jallon P. (1998). Idiopathic generalized epilepsy of late onset. Seizure.

[55] Marini C., King M.A., Archer J.S., Newton M.R., Berkovic S.F. (2003). Idiopathic generalised epilepsy of adult onset: clinical syndromes and genetics. J Neurol Neurosurg Psychiatry.

[56] Nicolson A., Chadwick D.W., Smith D.F. (2004). A comparison of adult onset and "classical" idiopathic generalised epilepsy. J Neurol Neurosurg Psychiatry.

[57] Song X., Xia Z., Martinez D., Xu B., Spritzer Z., Zhang Y. (2025). Independent genetic strategies define the scope and limits of CDKL5 deficiency disorder reversal. Cell Rep Med.

[58] Saby J.N., Mulcahey P.J., Zavez A.E., Peters S.U., Standridge S.M., Swanson L.C. (2022). Electrophysiological biomarkers of brain function in CDKL5 deficiency disorder. Brain Commun.

[59] Mangatt M., Wong K., Anderson B., Epstein A., Hodgetts S., Leonard H. (2016). Prevalence and onset of comorbidities in the CDKL5 disorder differ from Rett syndrome. Orphanet J Rare Dis.

[60] Bahi-Buisson N., Nectoux J., Rosas-Vargas H., Milh M., Boddaert N., Girard B. (2008). Key clinical features to identify girls with CDKL5 mutations. Brain.

[61] Cao L., Zhang X., Lou T., Ma J., Wang Z., Kim S. (2025). Cdkl5 knockout mice recapitulate sleep phenotypes of CDKL5 deficient disorder. Int J Mol Sci.

[62] Kim J.Y., Bai Y., Jayne L.A., Cianciolo R.E., Bajwa A., Pabla N.S. (2020). Involvement of the CDKL5-SOX9 signaling axis in rhabdomyolysis-associated acute kidney injury. Am J Physiol Ren Physiol.

