# Supplementary material for: Safety and efficacy of Igk-TATk-CDKL5 gene therapy in mosaic CDKL5 deficiency
Source: Neurotherapeutics. 2025 Sep 2;22(6):e00727. doi: 10.1016/j.neurot.2025.e00727 (PMC12664459; doi:10.1016/j.neurot.2025.e00727)
Supplement: Multimedia component 1 [file mmc1.pdf]

**Primary antibodies**			
**Target**	**Description**	**Dilution**	**Manufacturer**
AIF-1	Rabbit polyclonal	IHC 1:300	Thermo Fisher Scientific
PSD-95	Rabbit polyclonal	IHC 1:200	Abcam
HA	Rabbit monoclonal	IHC 1:500	Cell signalling Technology
VGlut1	Goat polyclonal	IHC 1:300	Synaptic system
P-γH2AX-Ser139	Rabbit polyclonal	IHC 1:500	Abcam
GFAP	Rabbit polyclonal	IHC 1:400	Abcam
CDKL5	Sheep polyclonal	WB 1:500	University of Dundee
P-EB2	Rabbit polyclonal	WB 1:1000	Covalab
EB2	Rabbit polyclonal	WB 1:2000	Abcam
GAPDH	Rabbit polyclonal	WB 1:5000	Sigma Aldrich
**Secondary antibodies**			
**Description**		**Dilution**	**Manufacturer**
Donkey anti-Rabbit IgG Cy3-conjugated		IHC 1:200	Jackson ImmunoResearch
Donkey anti-Goat IgG Cy3-conjugated		IHC 1:200	Jackson ImmunoResearch
Goat anti-Sheep IgG HRP-conjugated		WB 1:5000	Jackson ImmunoResearch
Goat anti-Rabbit IgG HRP-conjugated		WB 1:5000	Jackson ImmunoResearch

Supplementary Table 1. List of primary and secondary antibodies. WB: western blotting, IHC: immunohistochemistry.

Wake-sleep behavior				
**State**		**Measure (% time)**		
		+/+	+/-	**+/- GT**
Wakefulness	light	36 ± 1	27 ± 2**	26 ± 2***
	dark	61 ± 2	62 ± 3	54 ± 3
NREMS	light	53 ± 1	60 ± 2**	61 ± 2**
	dark	33 ± 2	30 ± 2	38 ± 3
REMS	light	8 ± 1	9 ± 1	8 ± 0.4
	dark	3 ± 0.2	4 ± 0.2	4 ± 0.3
**Sleep fragmentation**		18 ± 4	20 ± 5	20 ± 7

Supplementary Table 2. Effect of TATk-CDKL5 gene therapy on wake-sleep behavior in heterozygous *Cdkl5* female mice. The percentage of time spent in wakefulness, NREMS, and REMS states was evaluated over a 24-hour period, along with the sleep fragmentation index (calculated as the number of awakenings per total sleep time over 48 hours), in wild-type (+/+; n = 14), heterozygous *Cdkl5* (+/−; n = 11) mice, and heterozygous *Cdkl5* mice treated with TATk-CDKL5 gene therapy (+/− GT; n = 10). Values are represented as mean ± SEM. * $p < 0.05$, ** $p < 0.01$. Tukey's test after One-way ANOVA.
