# Supplementary material for: Safety and efficacy of Igk-TATk-CDKL5 gene therapy in mosaic CDKL5 deficiency
Source: Neurotherapeutics. 2025 Sep 2;22(6):e00727. doi: 10.1016/j.neurot.2025.e00727 (PMC12664459; doi:10.1016/j.neurot.2025.e00727)
Supplement: Multimedia component 2 [file mmc2.pdf]

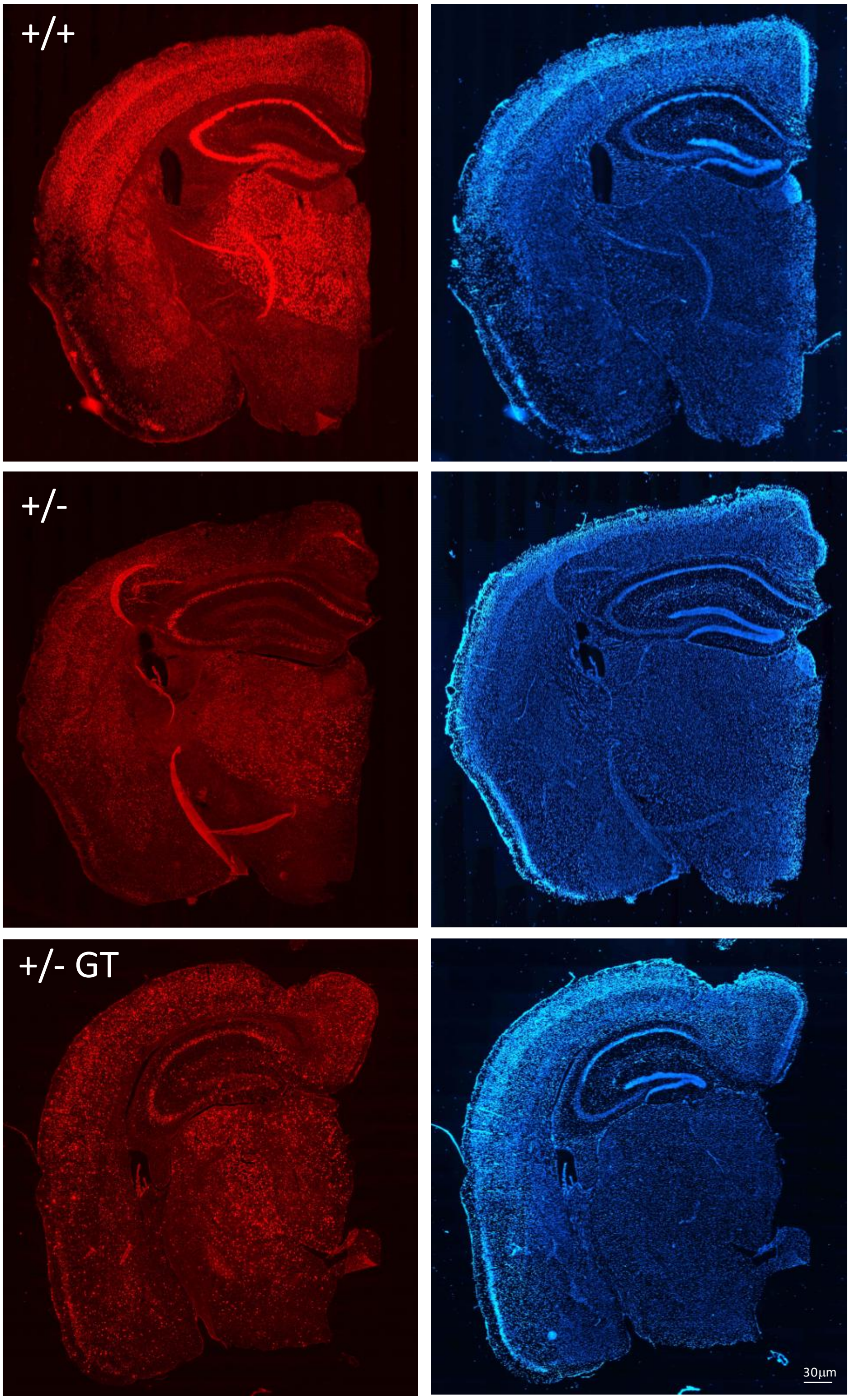

Supplementary Fig. 1. ***CDKL5*** **mRNA expression in the brain of** ***Cdkl5*** **+/− mice following gene therapy.**

Representative coronal brain sections with fluorescence in situ hybridization (ISH) for *CDKL5* mRNA (red, on the left) and nuclei counterstained with DAPI (blue, on the right) of a wild-type (+/+) and a *Cdkl5* +/− mouse, and of an AAVPHP.B_Igk-TATk-CDKL5 treated (+/− GT) mouse. Scale bar = 30 µm.

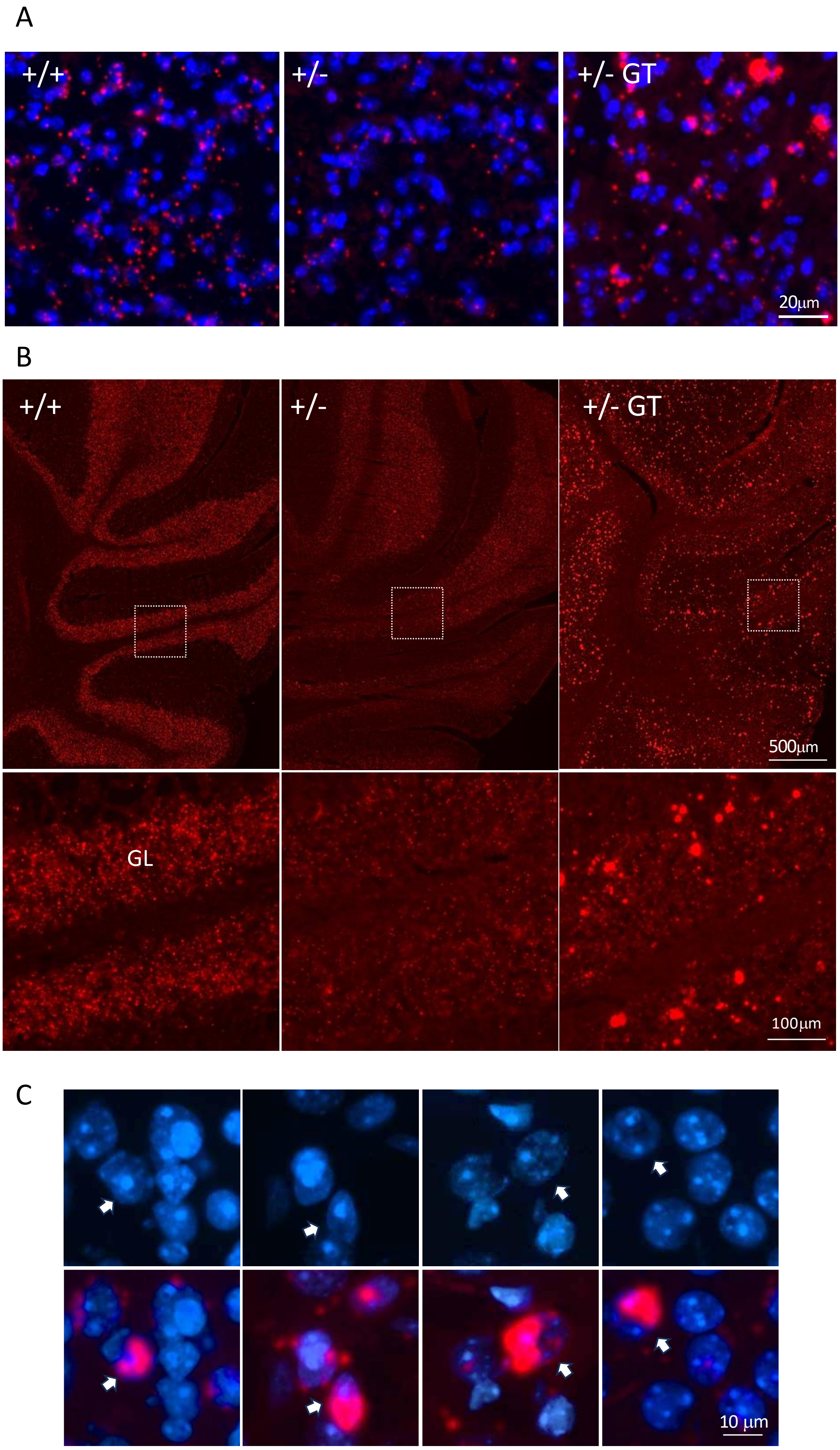
A
+/+
+/-
+/- GT
20µm
B
+/+
+/-
+/- GT
500µm
GL
100µm
C
10 µm

Supplementary Fig. 2. *CDKL5* mRNA expression in the brain of *Cdkl5* +/− mice following gene therapy.

A: Representative fluorescence images of brainstem sections processed for in situ hybridization (ISH) for *CDKL5* mRNA (red) of one animal from each experimental group. Nuclei were counterstained with DAPI (blue). Scale bar = 20 µm. **B:** Upper panels show representative images of cerebellar sections with fluorescence in situ hybridization (ISH) for *CDKL5* mRNA (red) of a wild-type (+ /+) and *Cdkl5* +/− mouse, and of a *Cdkl5* +/− mouse treated with the gene therapy AAVPHP.B_Igk-TATk-CDKL5 (+/− GT) vector. Scale bar = 500 µm. The dotted boxes in the upper panels indicate cerebellar granule cell layers shown in magnification in lower panels. Scale bar = 100 µm. Abbreviations: GL = granular layer of the cerebellar cortex. **C:** Fluorescence in situ hybridization (ISH) for *CDKL5* mRNA in cortical sections of a *Cdkl5* +/− mouse treated with the gene therapy AAVPHP.B_Igk-TATk-CDKL5 (+/− GT) vector. Upper panels show magnification of the nuclear morphology (DAPI staining in blue) of cells overexpressing *CDKL5* mRNA, highlighted by a white arrow. The lower panels show in situ hybridization (ISH) signals for *CDKL5* mRNA (red) in the same cells. Nuclei were counterstained with DAPI.

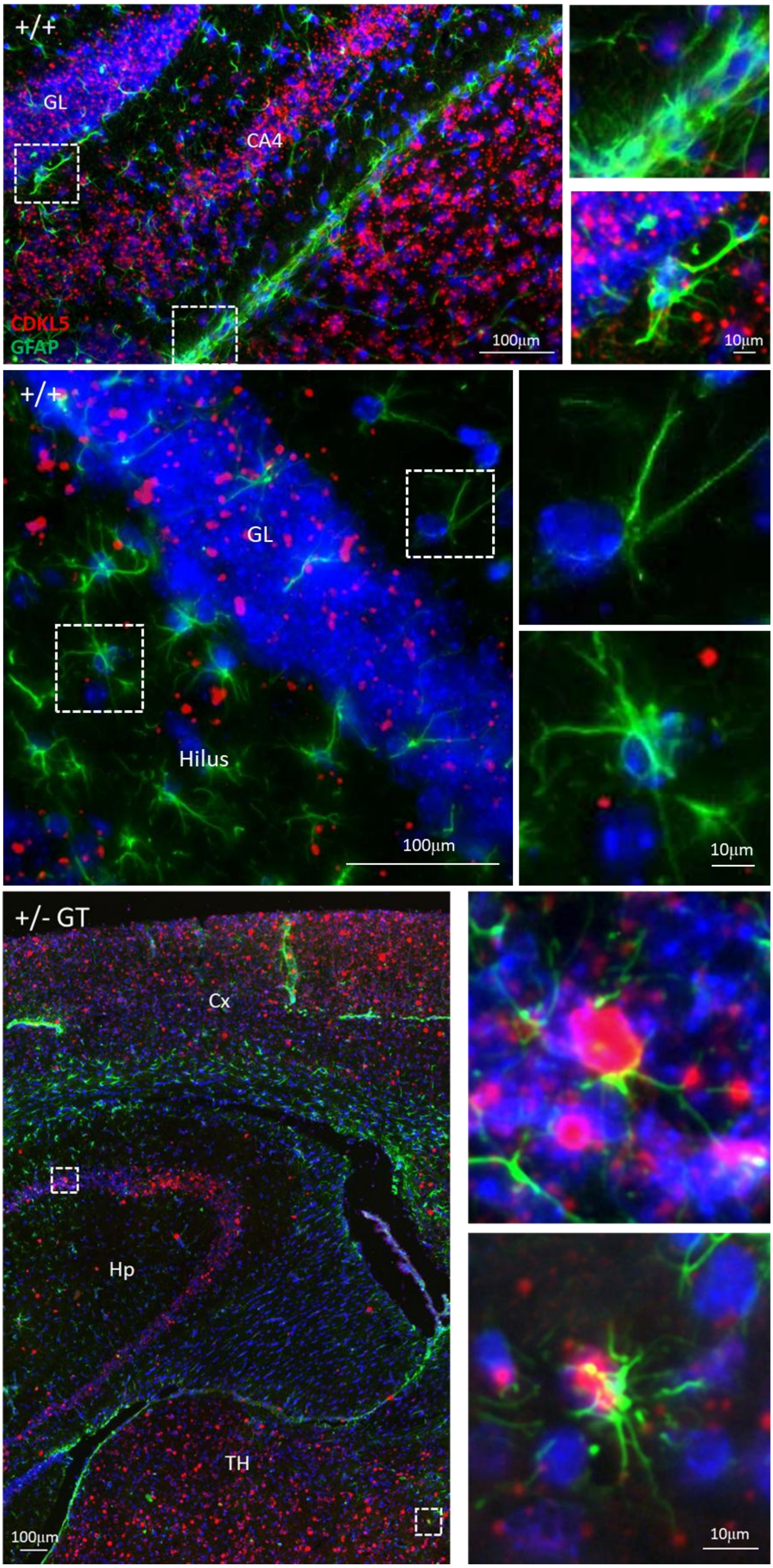
+/+
GL
CA4
CDKL5
GFAP
100µm
10µm
+/+
GL
Hilus
100µm
10µm
+/- GT
Cx
Hp
TH
100µm
10µm

Supplementary Fig. 3. *CDKL5* mRNA and Glial Fibrillary Acidic protein expression in the brain of *Cdkl5* +/− mice following gene therapy.

Fluorescence in situ hybridization (ISH) for *CDKL5* mRNA (CDKL5) combined with fluorescence immunolabeling for Glial Fibrillary Acidic Protein (GFAP) in mouse brain sections of a middle-aged wild-type (+/+) mouse and a *Cdkl5* +/− mouse treated with the gene therapy AAVPHP.B_Igk-TATk-CDKL5 (+/− GT) vector four months post-injection. The images illustrate the localization of *CDKL5* mRNA, assessed by in situ hybridization (ISH) using a CDKL5 probe (red), alongside the GFAP protein localization, identified through immunohistochemistry with an anti-GFAP antibody (green). Nuclei were counterstained with DAPI. The left panels present low magnification images showing the distribution of *CDKL5* mRNA and GFAP in brain of a wild-type (+/+) or a gene therapy-treated mouse (+/− GT), scale bar = 100 μm. Dotted boxes indicate regions which are magnified in the right panels (scale bar = 10 μm). Abbreviations: GL = granular layer of the hippocampal dentate gyrus, CA4 = hippocampal CA4 field, Cx = cortex, Hp = hippocampus, TH = thalamus.

A

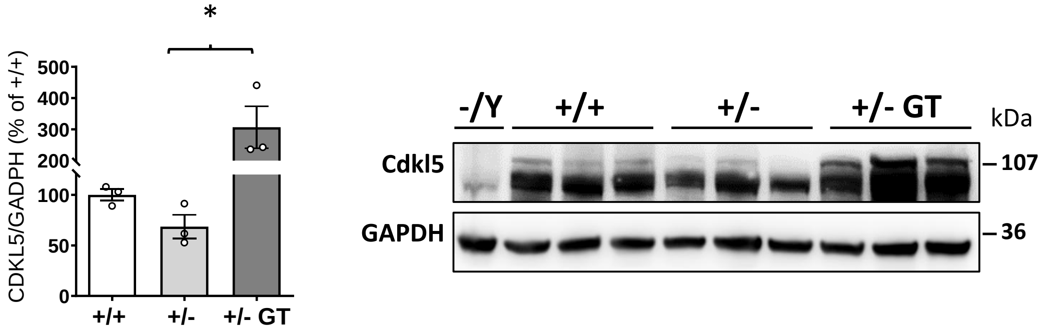
*
CDKL5/GADPH (% of +/+)
500
400
300
200
100
50
0
+/+
+/-
+/- GT
-/Y
+/+
+/-
+/- GT
kDa
Cdkl5
GAPDH
107
36

B

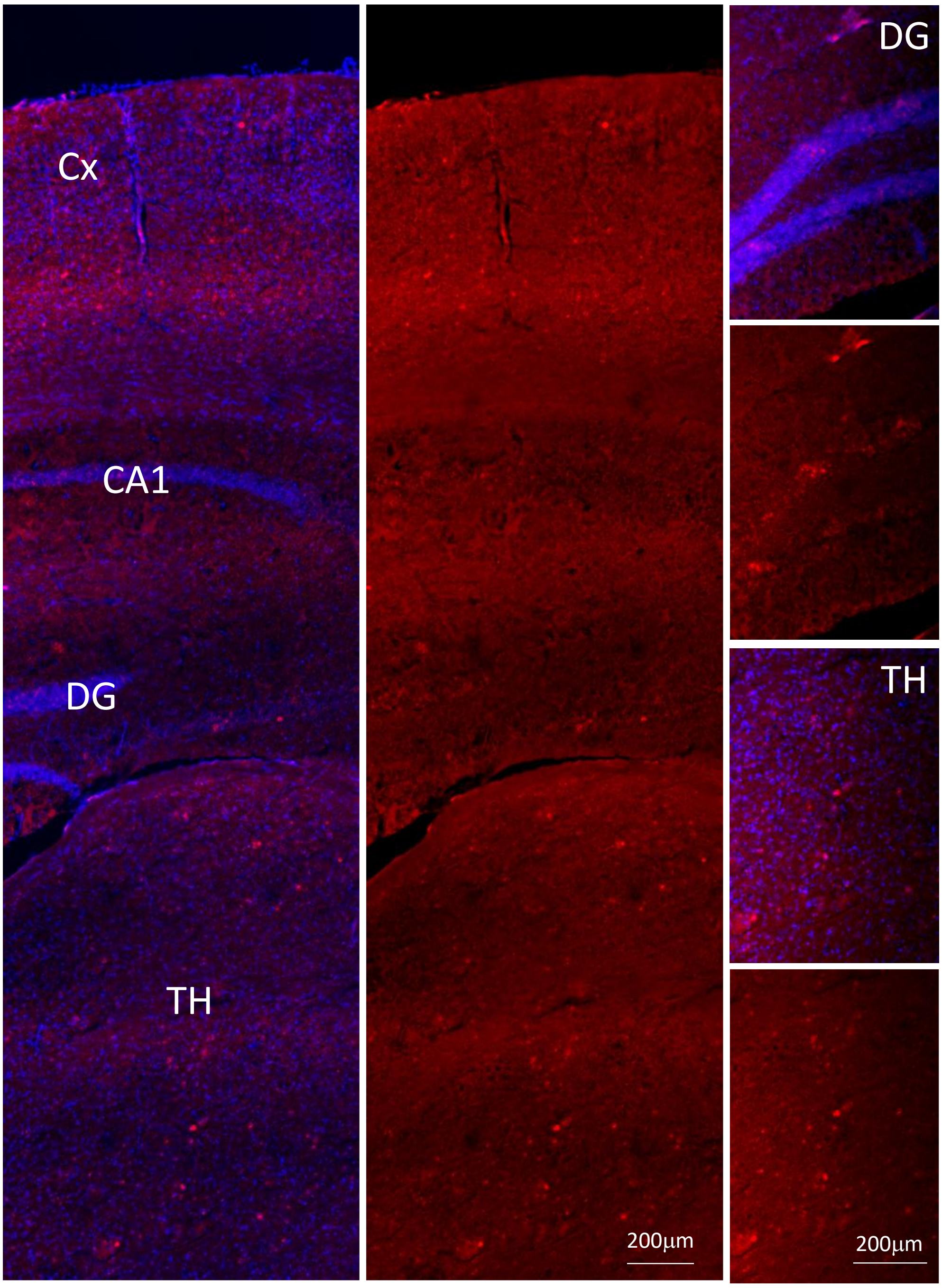
Cx
CA1
DG
TH
DG
TH
200µm
200µm

Supplementary Fig. 4. CDKL5 protein expression in the brain of *Cdkl5* +/− mice following gene therapy.

A: Western blot analysis of CDKL5 expression in somatosensory cortex homogenates from wild-type mice (+/+; n = 3), *Cdkl5* +/− mice (+/−; n = 3), and *Cdkl5* +/− mice treated with the gene therapy AAVPHP.B_Igk-TATk-CDKL5 (+/− GT; n = 3) vector. Histogram on the left shows CDKL5 protein levels normalized to GAPDH. Data are expressed as a percentage of the untreated wild-type condition. Example of immunoblots from three animals of each experimental group on the right. The first lane shows the absence of Cdkl5 expression in protein extracts from one hemizygous *Cdkl5* knockout male mouse (-/Y). Values represent means ± SEM. * $p < 0.05$, Fisher's LSD test after one-way ANOVA. **B:** Representative fluorescence images of a brain section processed for HA immunostaining showing the levels of TATk-CDKL5 expression in a *Cdkl5* +/− mouse treated with the gene therapy AAVPHP.B_Igk-TATk-CDKL5 vector (+/− GT). Abbreviations: Cx = somatosensory cortex, CA1 = hippocampal CA1 field, DG = dentate gyrus, TH = thalamus.

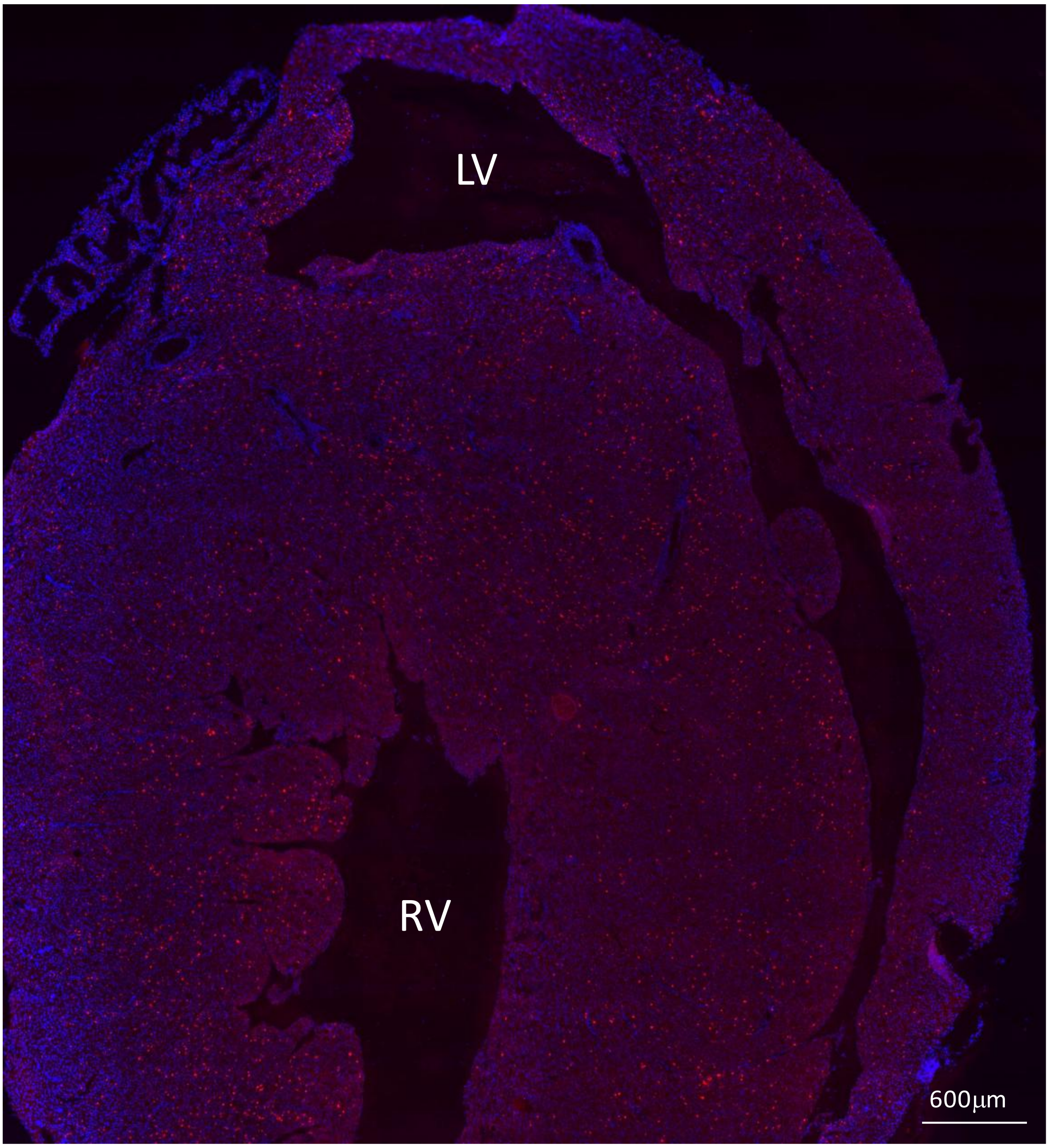

Supplementary Fig. 5.
Representative fluorescence image of a cardiac section processed for in situ hybridization (ISH) for *CDKL5* mRNA (red) of a *Cdkl5* +/+ mouse treated with the gene therapy AAVPHP.B_Igk-TATk-CDKL5 vector. Nuclei were counterstained with DAPI (blue). Scale bar = 600 µm. Abbreviations: RV = right ventricle, LV = left ventricle.
